# The Human DNA glycosylases NEIL1 and NEIL3 Excise Psoralen-Induced DNA-DNA Cross-Links in a Four-Stranded DNA Structure

**DOI:** 10.1038/s41598-017-17693-4

**Published:** 2017-12-12

**Authors:** Peter R. Martin, Sophie Couvé, Caroline Zutterling, Mustafa S. Albelazi, Regina Groisman, Bakhyt T. Matkarimov, Jason L. Parsons, Rhoderick H. Elder, Murat K. Saparbaev

**Affiliations:** 1 Biomedical Research Centre, Cockcroft Building, University of Salford, Salford, M5 4NT UK; 2 Ecole Pratique des Hautes Etudes, Paris, France Laboratoire de Génétique Oncologique EPHE, INSERM U753, Villejuif, France; Faculté de Médecine, Université Paris-Sud, Kremlin-Bicêtre, France; 3 Groupe «Réparation de l’ADN», Equipe Labellisée par la Ligue Nationale Contre le Cancer, CNRS UMR8200, Université Paris-Sud, Gustave Roussy Cancer Campus, F-94805 Villejuif Cedex, France; 4 National Laboratory Astana, Nazarbayev University, Astana, 010000 Kazakhstan; 5 Cancer Research Centre, Department of Molecular and Clinical Cancer Medicine, University of Liverpool, 200 London Road, Liverpool, L3 9TA UK

## Abstract

Interstrand cross-links (ICLs) are highly cytotoxic DNA lesions that block DNA replication and transcription by preventing strand separation. Previously, we demonstrated that the bacterial and human DNA glycosylases Nei and NEIL1 excise unhooked psoralen-derived ICLs in three-stranded DNA *via* hydrolysis of the glycosidic bond between the crosslinked base and deoxyribose sugar. Furthermore, NEIL3 from *Xenopus laevis* has been shown to cleave psoralen- and abasic site-induced ICLs in *Xenopus* egg extracts. Here we report that human NEIL3 cleaves psoralen-induced DNA-DNA cross-links in three-stranded and four-stranded DNA substrates to generate unhooked DNA fragments containing either an abasic site or a psoralen-thymine monoadduct. Furthermore, while Nei and NEIL1 also cleave a psoralen-induced four-stranded DNA substrate to generate two unhooked DNA duplexes with a nick, NEIL3 targets both DNA strands in the ICL without generating single-strand breaks. The DNA substrate specificities of these Nei-like enzymes imply the occurrence of long uninterrupted three- and four-stranded crosslinked DNA-DNA structures that may originate *in vivo* from DNA replication fork bypass of an ICL. In conclusion, the Nei-like DNA glycosylases unhook psoralen-derived ICLs in various DNA structures *via* a genuine repair mechanism in which complex DNA lesions can be removed without generation of highly toxic double-strand breaks.

## Introduction

Due to their high cytotoxicity, DNA crosslinking agents such as mitomycin C, cisplatin and psoralens are widely used against hyperplasic diseases such as cancer and psoriasis^1,2^. Furanocoumarins (psoralens) require UVA-photoactivation following DNA intercalation to chemically react with both cellular DNA *in vivo* and naked DNA *in vitro* ^3^. Psoralen derivatives 8-methoxypsoralen (8-MOP) and 4,5′,8-trimethylpsoralen (HMT/trioxsalen) are asymmetric, planar, tricyclic compounds that intercalate into the DNA duplex near pyrimidines, preferentially at 5′-TpA sites. Upon photoactivation, 8-MOP and HMT primarily photoalkylates DNA by cycloaddition to the 5,6-double bond of a thymidine generating monoadducts (MA) with either the 4′,5′-double bond of the furan (MAf) or the 3,4-double bond of the pyrone (MAp) side of the psoralen^4^ (Supplementary Table S1). Absorption of a second photon by the MAf leads to formation of a 5,6-double bond adduct with a flanking thymine in the complementary strand, thus generating an interstrand DNA cross-link (ICL)^5^. HMT induces a higher yield of ICLs in duplex DNA as compared to 8-MOP. While both psoralens produce MAs and ICLs, the latter class of damage appears to have a more severe biological effect^3^.

DNA glycosylases accomplish the removal of chemically modified bases in DNA in the first step of base excision repair (BER) by cleaving the glycosidic bond between the deoxyribose sugar and the base^6^. DNA glycosylases are divided between mono- and bifunctional enzymes based on their mechanism of action. The mono-functional DNA glycosylases cleave the *N*-glycosidic bond, releasing the modified base and generating an apurinic/apyrimidinic (AP) site^7^. The bi-functional glycosylases not only cleave the *N*-glycosidic bond, but also in a concerted manner cleave the phosphodiester bond 3′ to the resulting AP site by a β or β-δ elimination mechanism (AP lyase activity), generating a single-strand break with 3′-phosphate/phosphoaldehyde and 5′-phosphate ends at the single-nucleotide gap^7–9^. Three Nei-like (NEIL) DNA glycosylases are present in mammalian cells; these proteins show structural homology to the Fpg and Nei proteins of *Escherichia coli* and remove oxidised bases from DNA^10^. While NEIL1 and NEIL3 appear to be cell cycle regulated, expression peaking in S phase and late S/G2 respectively, NEIL2 is constitutively expressed throughout the cell cycle^11,12^. All three have DNA glycosylase activity with an unusual preference for single-stranded DNA and other DNA open structures found during DNA replication and transcription. Both NEIL1 and NEIL2 excise the modified base and cleave the resulting AP site in DNA *via* β/δ-elimination in a highly concerted manner^13,14^. In contrast, NEIL3 exhibits a non-concerted action with base excision being more efficient than AP site cleavage activity^15^. Mouse and human NEIL3 incise an AP site *via* β-elimination with very low efficiency, thus they can be considered essentially as mono-functional DNA glycosylases by their mode of operation^15,16^. NEIL3 is perhaps the most intriguing of the three due, (*i*) to its larger structure that includes an extended C-terminal domain containing additional zinc finger motifs, (*ii*) the replacement of the usual proline residue as the nucleophile with valine and, (*iii*) its restricted expression pattern in mammalian cells^10^. Interestingly, the replacement of Valine-2 by Proline in NEIL3 restores concerted action since NEIL3-V2P mutant exhibits coupled base excision and strand incision activities^15^.

Genetic and biochemical evidences suggest that in vertebrates, repair of ICLs is linked to DNA replication and proceeds via induction of a double-strand break (DSB) as a result of the unhooking via dual incisions on either side of the ICL by the scaffolding protein SLX4 and structure-specific endonuclease XPF/ERCC1^17,18^. The resulting unhooked ICL swings free of the duplex exposing a single-stranded gap. Replication bypass of the gap can be catalyzed by translesion synthesis (TLS) specific DNA polymerases yielding a three-stranded DNA repair intermediate composed of a short oligomer covalently bound to the duplex. Surprisingly in *Xenopus* egg extracts the remaining crosslinked fragment persist and does not interfere with the repair of DSB by Rad51-catalyzed homologous recombination (HR)^19^. However, in living cells the adduct is most likely removed either through BER or nucleotide excision repair (NER) pathways. Recent progress in understanding the repair mechanism of ICLs in vertebrates has revealed the existence of an incision-independent repair mechanism. Reconstitution of the repair of plasmids containing a single ICL in cell-free extracts from *Xenopus* eggs showed that ICL repair is coupled to DNA replication and involves convergence of two replication forks on the lesion with the formation of an X-shaped DNA structure^20^. The authors proposed that ICL repair requires the convergence of two forks on the lesion since, when only one fork was stalled at the ICL in egg extracts, no ICL repair was initiated^21^. Similarly, Huang and colleagues investigated the collision of replication forks with fluorescently marked psoralen ICLs in mammalian cells using DNA combing^22^. They observed that during S phase the majority of ICLs (around 60%) are processed through a replication-traverse pathway, in which the ICLs are left unrepaired, but are traversed by the replication machinery to allow DNA synthesis to resume on the other side of fork. The authors proposed that the unrepaired ICLs are subsequently removed during a post-replication repair process by NER or BER without the generation of DSBs^22^. Conversely, a minority of ICLs (around 20%) block progression of replication forks, in forms of either single-fork collision or dual-fork collision (in which two converging forks collide with the same ICL)^22^. Nevertheless, in both scenarios of fork traverse and dual fork convergence, a similar X-shaped DNA structure is generated around the ICL, which is critical to initiate ICL repair^23^.

It was thought that in mammalian cells, bulky DNA lesions such as ICLs were eliminated mainly in an incision-dependent pathway that proceeds *via* generation of a DSB^24^. Recently, attention has focused on the ability of Nei-like DNA glycosylases to resolve ICLs in DNA in an incision-independent pathway^25^. Our previous work was the first to show involvement of bacterial Nei and human NEIL1 in the repair of psoralen adducted DNA, resolving MAs in dsDNA and ICLs in a three-stranded DNA context^26^. Furthermore, sensitivity to 8-MOP and UVA radiation was observed after knockdown of NEIL1 and AP endonuclease 1 (APE1) in HeLa cells, thus further implicating BER in the repair of ICLs^26,27^. Subsequently, Semlow and colleagues have shown that NEIL3 from *Xenopus laevis* can also repair psoralen and abasic site ICLs in DNA in X-shaped dsDNA structures, suggesting that this activity may be a principal role of NEIL3 in rapidly dividing cells^28^. Here, we constructed three- and four-stranded DNA structures containing a single psoralen-derived ICL that mimic intermediates of ICL replication and repair, and then examined their repair by the Nei-like DNA glycosylases, Nei, NEIL1 and NEIL3. Potential involvement of FANCM mediated replication fork traverse in the regulation of BER is discussed. The present study provides new biochemical and genetic insights into the molecular mechanism of ICL repair.

## Results

### Purification and characterization of human NEIL3 proteins

Here we cloned and expressed in *E. coli* the wild-type full-length NEIL3 with a C-terminal His tag referred to as NEIL3^FL^ and also constructed two C-terminal truncation mutants of NEIL3 fused with a C-terminal His tag referred to as NEIL3^Cat^ comprising amino acids 1–281 (containing the Fpg/Nei and H2TH domains) and NEIL3^Trun^ comprising amino acids 1–348 (containing in addition the RanBP zinc finger domain) (Supplementary Fig. S1). Truncated versions of NEIL3 were more stable than the full-length wild-type protein and we were able to over-express and purify them in a more active form than the full-length version. To ensure that the NEIL3-catalyzed DNA repair activities are not due to trace contamination by host DNA glycosylases, we changed an absolutely conserved catalytic lysine residue to alanine in NEIL3^Cat^ resulting in a single substitution K81A and purified it using the same scheme as for the wild-type protein.

We tested enzymatic activities of the purified human NEIL3^Cat^, NEIL3^Cat^-K81A, NEIL3^Trun^, NEIL3^FL^, NEIL1 and *E. coli* Nei proteins on 17 mer single-stranded (ss) and duplex (ds) oligonucleotides containing a single Sp residue. As NEIL3 is essentially a mono-functional DNA glycosylase, to reveal the presence of non-cleaved AP sites after reaction, the samples were treated with light piperidine (10% (v/v) piperidine at 37 °C for 30 min). Analysis of the reaction products on 20% denaturing polyacrylamide gel electrophoresis (PAGE) revealed that all three NEIL3 proteins excise Sp in ssDNA in an enzyme concentration dependent manner (Supplementary Fig. S2). Nevertheless, truncated versions of the human DNA glycosylase, NEIL3^Cat^ and NEIL3^Trun^, excised Sp with 3–4 fold higher efficiencies than NEIL3^FL^. As expected, no cleavage was observed when Sp substrates were incubated with the NEIL3^Cat^-K81A mutant, indicating that our NEIL3 preparations are not contaminated with bacterial DNA glycosylases. These results indicate that the NEIL3 protein preparations are active and confirm that NEIL3 is primarily a monofunctional DNA glycosylase on these substrates^15,16^.

### Construction and characterization of three- and four-stranded DNA structures containing a single ICL

**Figure 1:**
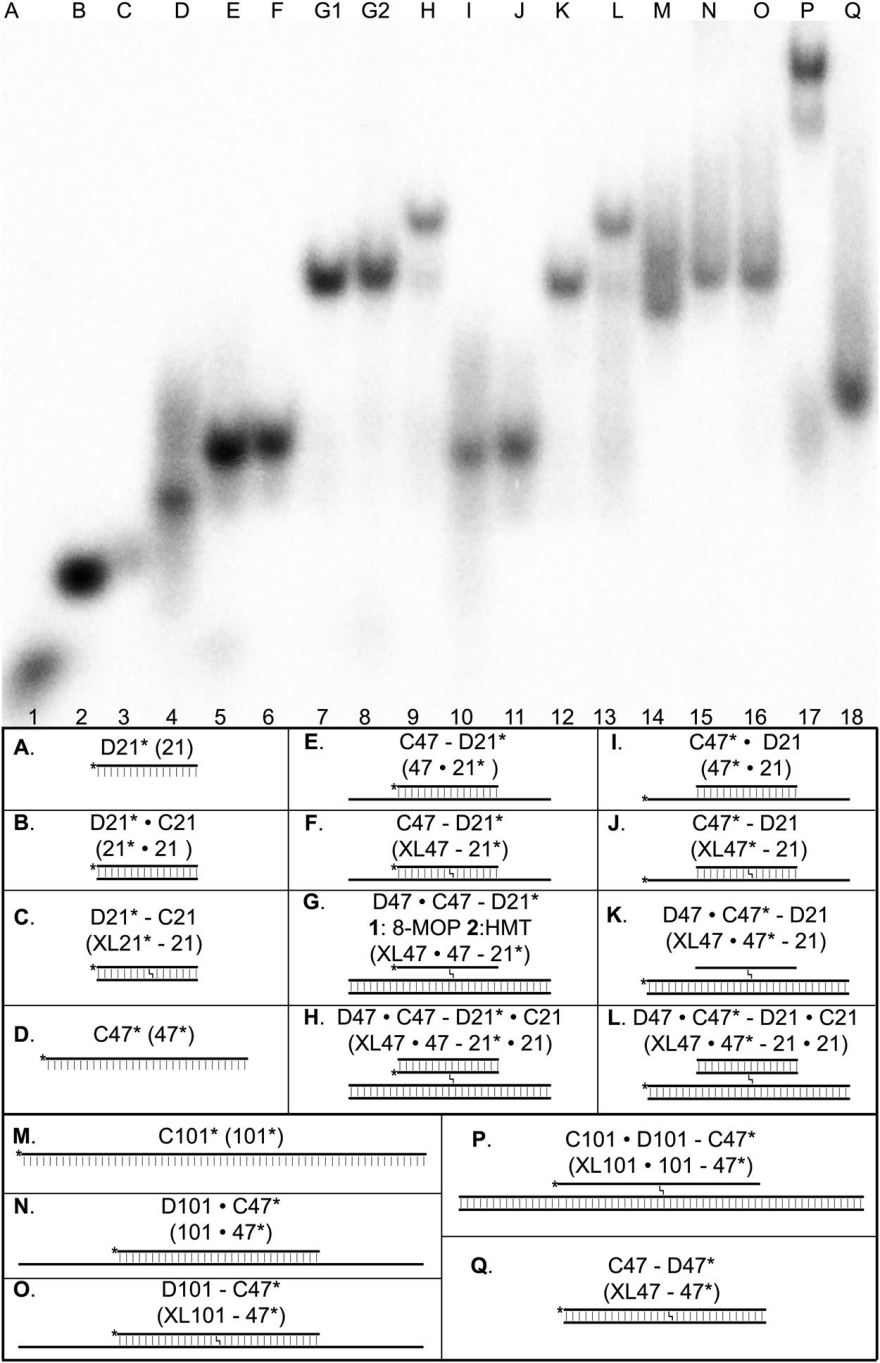
Non denaturing PAGE analysis of the DNA structures containing single ICL used in DNA glycosylase activity assays. 5′-[^32^P]-labelled oligonucleotide strands are denoted by “*”, the presence of a crosslink is denoted by “-” and normal oligonucleotide base complementation is indicated by “∙”.

To further characterize DNA substrate specificities of the Nei, NEIL1 and NEIL3 DNA glycosylases, we constructed and characterized long, three- and four-stranded DNA structures containing a single psoralen-derived ICL. These crosslinked DNA structures mimic the products of DNA translesion synthesis bypass of an ICL site in duplex DNA. The migration pattern of all DNA structures was first analyzed by non-denaturing PAGE (Fig. 1). Oligonucleotide sequences used in this work are shown in Supplementary Table S2. To start the construction, the 5′-[^32^P]-labelled single-stranded 21 and 47 mer DNA oligonucleotides, referred to as D21 and C47, respectively (containing a single 5′-TpA site at position 9 and 24, respectively), were annealed to the partially complementary 21, 47 and 101 mer oligonucleotides, referred as C21/D21, D47 and D101, respectively (containing a single 5′-TpA site at position 12/9, 23 and 49, respectively), to obtain non-covalent D21*∙C21, D21*∙C47/C47*∙D21 and C47*∙D101 duplex oligonucleotides (“*”denotes the 5′-[^32^P]-labelled oligonucleotide), respectively (Fig. 1, panels E, I and N). The oligonucleotide duplexes were exposed to either HMT + UVA or 8-MOP + UVA treatments and the resulting 5′-[^32^P]-labelled crosslinked DNA duplexes XL21-21, XL47-21 and XL101-47 were purified by denaturing PAGE (Fig. 1, panels F, J and O). Next, the purified XL47-21 and XL101-47 oligonucleotides containing a single ICL were hybridized to complementary D47 and C101 DNA oligonucleotides to obtain 5′-[^32^P]-labelled three-stranded DNA structures, XL47∙47-21 and XL101∙101-47 (Fig. 1, panels G, K, O and P). Finally, the three-stranded XL47∙47-21 oligonucleotides were hybridized to C21, to obtain the 5′-[^32^P]-labelled four-stranded DNA XL47∙47-21∙21 oligonucleotides (Fig. 1, panels H and L). Analysis of the migration pattern showed that the single-stranded oligonucleotides migrate faster than the duplex structures while the crosslinked duplexes migrate slightly slower than their non-crosslinked counterparts. The three-stranded crosslinked DNA structures XL47∙47-21 and XL101∙101-47 (lanes 7, 8, 12 and 17) migrate much slower than the respective crosslinked duplexes XL47-21 and XL101-47 (lanes 6, 11 and 16). And finally, the four-stranded crosslinked DNA structures XL47∙47-21∙21 (lanes 9 and 13) migrate even more slowly than their respective three-stranded crosslinked DNA structures XL47∙47-21 (lanes 7, 8 and 12). We observed smearing of DNA bands on gel electrophoresis corresponding to the D, I, M, N, O, P and Q substrates (lanes 4 and 14−17) which may be due to secondary structures formed by C47 oligonucleotide (lane 4). Taken together, the analysis of the migration patterns of crosslinked DNA duplexes on a non-denaturing gel suggest the formation of stable three- and four-stranded DNA structures.

### NEIL3 excises an unhooked ICL within a three-stranded DNA structure

**Figure 2:**
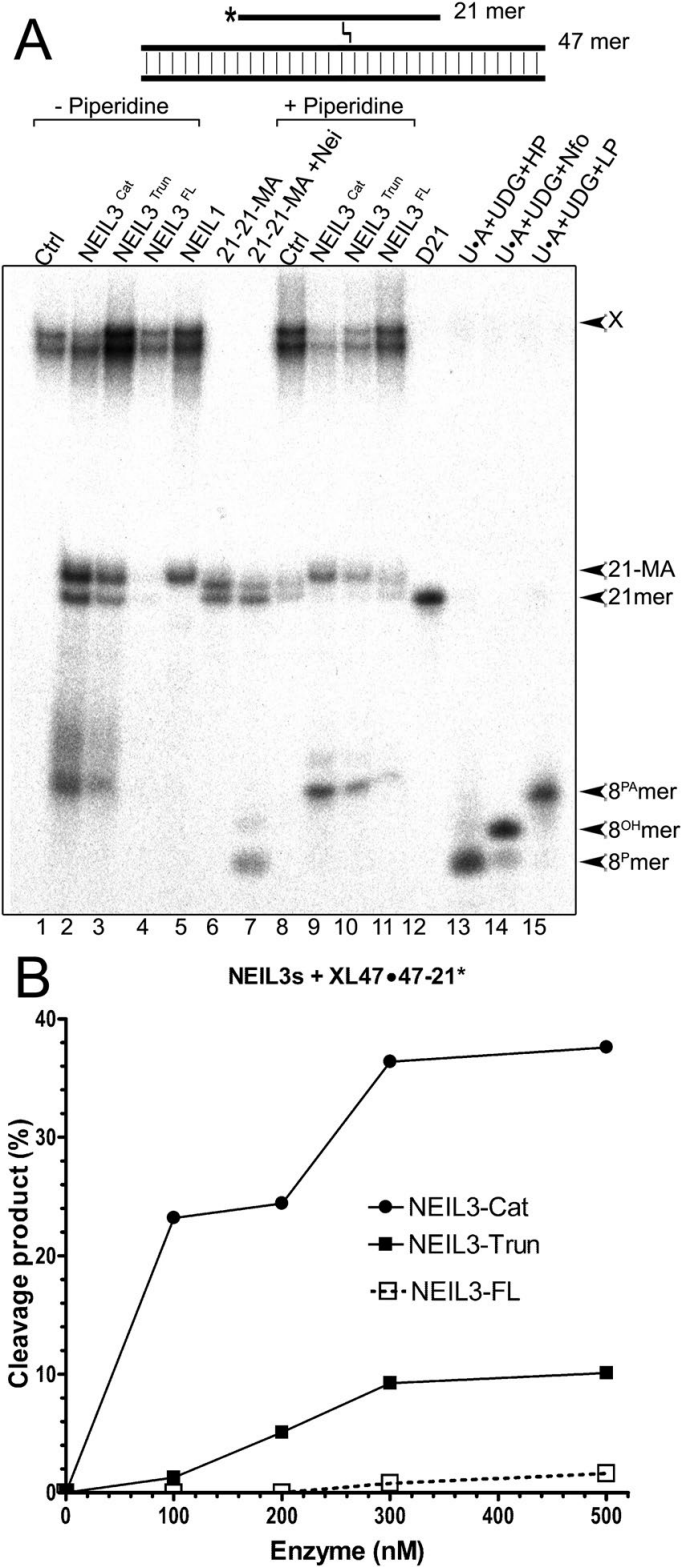
Action of Nei-like DNA glycosylases upon three-stranded DNA structure containing single HMT-derived ICL. (**A**) Denaturing PAGE analysis of the reaction products. 10 nM 5′-[^32^P]-labelled XL47∙47-21* was incubated for 1 hr at 37 °C either with the 500 nM NEIL3^Cat^, NEIL3^Trun^, NEIL3^FL^ or with 50 nM NEIL1 proteins. Lanes 1–7, no piperidine treatment; lane 1, control non-treated XL47∙47-21*; lanes 2–4, as 1 but with NEIL3s; lane 5, NEIL1; lane 6, 21 mer duplex containing MA; lane 7, as 6 but Nei; lanes 8–11, as 1–4 but treated with light piperidine; lane 12, 21 mer D21; lanes 13–15, 21 mer U∙A duplex treated with UDG/hot piperidine, UDG/Nfo and UDG/light piperidine, respectively. Substrate and cleavage products sizes are indicated to the right of the gel. “X”denotes substrate, “21-MA” denotes 21 mer fragment containing psoralen-derived MA, “21-mer” denotes size marker, “8 ^PA^”, “8^OH^” and “8^P^” denote 8 mer fragments containing 3′-terminal PA, OH and P, respectively. For details see Materials and Methods. (**B**) Graphical representation of NEIL3s protein concentration dependent activities upon three-stranded DNA structure.

Recently, it has been shown that *Xenopus* NEIL3 is able to excise thymine and adenine nucleotides crosslinked to a single-stranded oligonucleotide, suggesting that the NEIL3 homologs can remove psoralen-generated ICLs when present in DNA^28^. To examine whether human NEIL3 excises a psoralen-induced ICL in three-stranded DNA structures, we utilised the 5′-[^32^P]-labelled XL47∙47-21* and XL47∙47*−21 oligonucleotides (“*”denotes the 5′-[^32^P]-labelled strand) containing a 21 mer oligonucleotide crosslinked by HMT + UVA exposure to a 47 mer duplex (see Fig. 1, panels G2 and K). To identify DNA glycosylase cleavage products we prepared oligonucleotide size markers containing: (*i*) a single psoralen-derived thymine monoadduct (MA), for this, an HMT-crosslinked XL21*−21 and XL47*−21 duplexes were incubated under hot-alkali conditions^29^; (*ii*) 3′-terminal phosphate (P); (iii) 3′-terminal hydroxyl group (OH); and (iii) 3′-terminal α,β-unsaturated phosphoaldehyde (PA) (for details see Materials and Methods). Structure of DNA modifications used in this work are shown in Supplementary Table S1. As expected, incubation of the 5′-[^32^P]-labelled XL47∙47-21* (panel G2) with NEIL1 generated a 21 mer cleavage product (Fig. 2A, lane 5) that migrated slightly more slowly than the 21 mer with MA (21-MA) and regular 21 mer D21 oligonucleotide (lanes 6 and 12). This result suggests that NEIL1: (*i*) remove a long crosslinked 21 mer oligomer from the XL47∙47-21* structure *via* excision of the crosslinked thymine base in the 47 mer duplex oligonucleotide; and (*ii*). generate a 5′-[^32^P]-labelled 21 mer ssDNA fragment containing a deoxythymidine monophosphate (dTMP) nucleotide crosslinked to an extrahelical thymine residue (Supplementary Table S1) and non-labelled nicked 47 mer duplex oligonucleotide. However, no cleavage of the 21 mer excision product was observed after incubation with NEIL1 suggesting that these DNA glycosylases recognize crosslinked thymine only within 47 mer duplex DNA.

Incubation of the 5′-[^32^P]-labelled XL47∙47-21* (Fig. 1, panel G2) with NEIL3^Cat^ and NEIL3^Trun^ without piperidine treatment resulted in the appearance of 21 mer excision fragments with a smeared migration pattern below suggesting the unhooking of the ICL in XL47-21 and formation of an abasic site in the released 21 mer D21* oligonucleotide (Fig. 2A, lanes 2 and 3). Incubation with NEIL3^FL^ generated cleavage products but with very low efficiency compared to the truncated proteins (lane 4). Here we used piperidine treatment to cleave AP sites generated by NEIL3. It should be noted that piperidine is a highly efficient β-catalyst that cleaves AP sites in DNA either *via* β-elimination at 37 °C or *via* β-δ elimination reaction at 90 °C^30-32^. Light piperidine treatment of the NEIL3 reaction products resulted in the formation of an 8 mer cleavage product (lanes 9–11) that migrated similar to the 8 ^PA^ mer size marker (lane 15) but much more slowly than the 8^OH^ size marker (lane 14) and 8^P^ mer product generated by Nei acting upon the 21 mer duplex containing an HMT-derived MA (lane 7). However, piperidine treatment did not result in the disappearance of free 21 mer D21* oligonucleotide suggesting that it still contains psoralen-derived adduct (lanes 9–11). These results suggest that the human NEIL3 proteins excise a long 21 mer single-stranded oligomer crosslinked to duplex DNA in XL47∙47-21* by hydrolysing the glycosidic bond between the crosslinked thymine and deoxyribose of either the 5′-[^32^P]-labelled D21 oligonucleotide or non-labelled 47 mer C47∙D47 duplex. It should be noted that incubation of non-treated XL47∙47-21* in light piperidine resulted in partial decross-linking with the appearance of 21-MA and 21 mer fragments (lane 8). The HMT-induced ICLs can be cleaved and converted to mono-adducts by alkaline treatment and the efficiency of decross-linking depends on temperature and nature of the alkali used^29^.

To assess relative efficiencies of the NEIL3 proteins on ICLs, we examined enzyme concentration dependent cleavage of XL47∙47-21*. As shown in Fig. 2B, NEIL3^Cat^ was the most efficient ICL-excising DNA glycosylase, when compared to the other NEIL3 proteins, cleaving 38% of the DNA at the highest enzyme concentration. Whereas, NEIL3^Trun^ cleaved 10% and NEIL3^FL^ exhibited very weak activity, cleaving only 2% of DNA substrate under the same reaction conditions. Similar results were obtained when incubating the NEIL3 proteins with the 8-MOP-derived three-stranded XL47∙47-21* (Fig. 1, panel G1 and Supplementary Fig. S3) suggesting that NEIL3 can recognize ICLs generated by different psoralens. As expected, no cleavage products were observed when the 5′-[^32^P]-labelled XL47∙47-21* was incubated with increasing amounts of the catalytically deficient NEIL3^Cat^-K81A mutant with or without piperidine treatment (Supplementary Fig. S4). Thus, NEIL3 can target HMT and 8-MOP crosslinked thymine either in the unhooked single-stranded portion or in duplex DNA of the three-stranded DNA structure and generate a free ssDNA fragment and duplex DNA, each containing either an abasic site or dTMP crosslinked to an extrahelical thymine residue, respectively.

**Figure 3:**
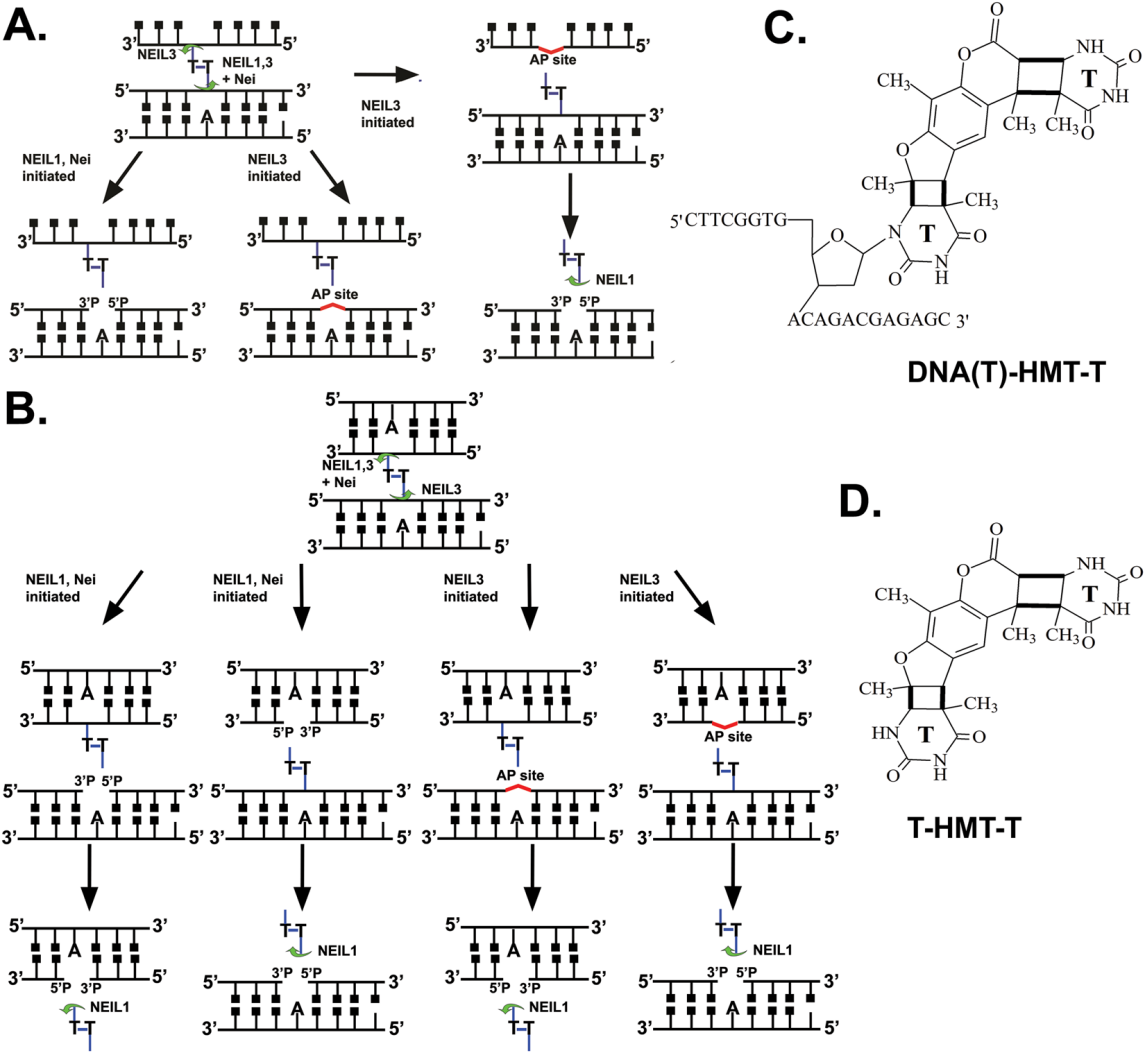
Schematic representation of the mechanisms of action of Nei-like DNA glycosylases on ICLs. (**A**) Mechanisms of action of Nei-like DNA glycosylases on three-stranded DNA structure containing single psoralen-derived ICL. For details see text. (**B**) Mechanisms of action of Nei-like DNA glycosylases on four-stranded DNA structure containing single psoralen-derived ICL. (**C**) Skeletal formula of 21 mer fragment containing single Thymidine nucleotide crosslinked to free Thymine base by HMT (DNA(T)-HMT-T), an excision product generated by Nei-like DNA glycosylases catalyzed repair of ICL in three-stranded DNA structure. (**D**) Skeletal formula of Thymine-HMT-Thymine cross-link (T-HMT-T), an excision product generated by Nei-like DNA glycosylases catalyzed repair of ICL in three- and four-stranded DNA structure.

To further substantiate the mechanisms of action of Neil-like DNA glycosylases, we used the three-stranded XL47∙47*−21 oligonucleotide substrate in which the 47 mer C47 oligonucleotide was 5′-[^32^P]-labelled (Fig. 1, panel K). Incubation of the 5′-[^32^P]-labelled XL47∙47*−21 with NEIL3s resulted in the formation of 47 mer and 23 ^PA^ mer cleavage products, whereas incubation with Nei generated a 23^P^ mer product only (Supplementary Fig. S5). Again these results suggest that in contrast to Nei, human NEIL3 can recognize crosslinked thymine residues in both the duplex and single-stranded oligonucleotide portions of the three-stranded DNA structure. Schematic representation of the mechanism of action of Nei-like DNA glycosylases upon three-stranded DNA structure is shown in Fig. 3A.

Next we examined whether the length of the crosslinked DNA fragment is critical for recognition of an ICL by the Nei-like DNA glycosylases. For this we constructed the three-stranded 5′-[^32^P]-labelled XL101∙101-47* oligonucleotide (“*”denotes the 5′-[^32^P]-labelled strand) containing a 47 mer C47 oligonucleotide crosslinked to a 101 mer C101∙D101 duplex (see Fig. 1, panel P). As expected, incubation of XL101∙101-47* with Nei, NEIL1 generated a ~47 mer cleavage product that migrated more slowly than the non-modified 47 mer C47 oligonucleotide (Supplementary Fig. S6). Whereas, incubation of the 5′-[^32^P]-labelled XL101∙101-47* with NEIL3^Cat^, NEIL3^Trun^ and NEIL3^FL^ followed by piperidine treatment resulted in the appearance of 47 mer and 23 mer cleavage products, the latter migrated more slowly than the 23 mer product generated by Nei when acting upon the MA containing 47 mer, C47*∙D47 duplex (Supplementary Fig. S6). These results are in agreement with the above data and suggests that Nei-like DNA glycosylases can remove a long crosslinked 47 mer oligomer from the XL101∙101-47* structure *via* excision of a crosslinked thymine either in the 101 mer duplex or in the 47 mer single-stranded oligomer.

### Nei-like DNA glycosylases cleave ICLs in a four-stranded DNA structure

**Figure 4:**
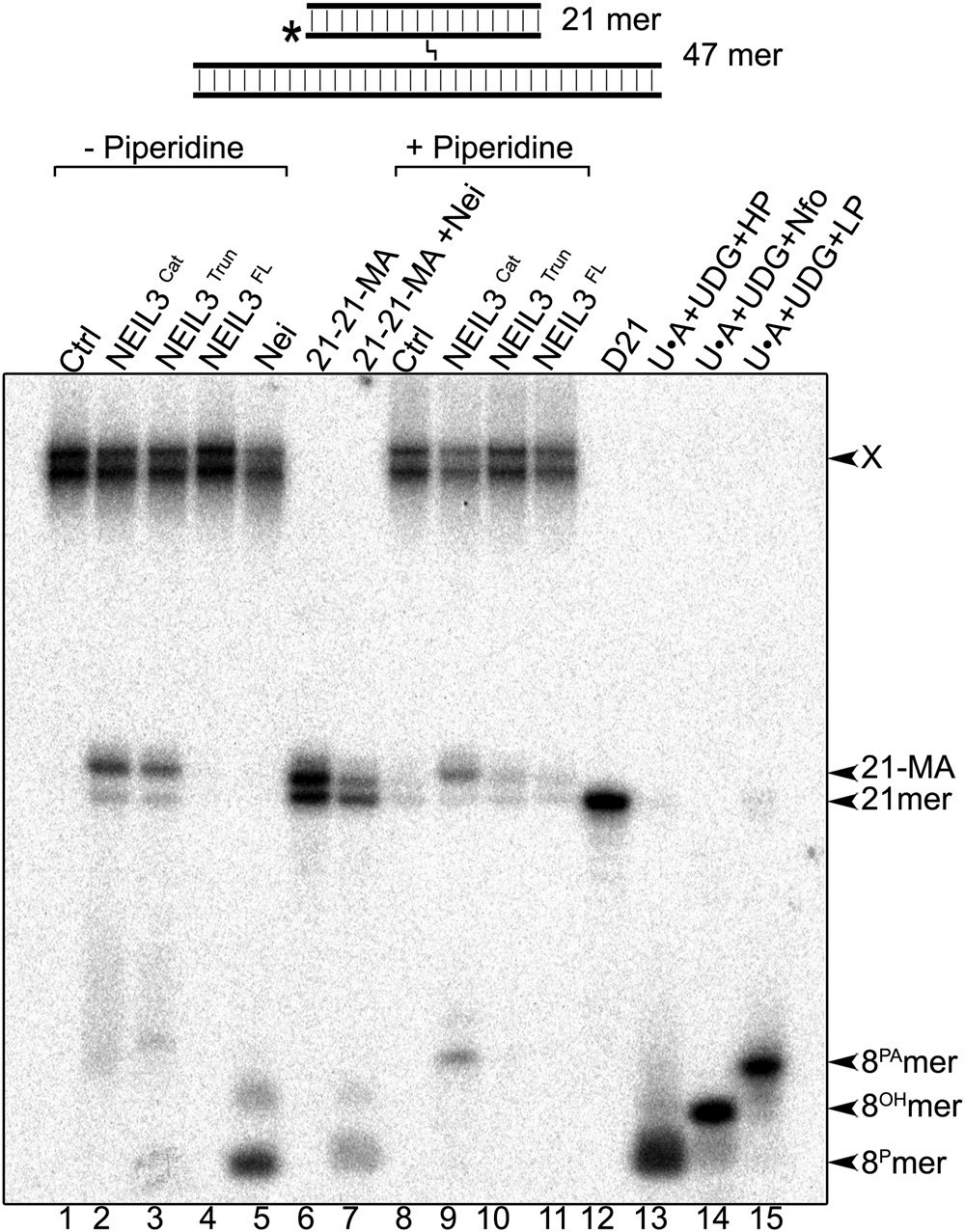
Action of Nei-like DNA glycosylases upon four-stranded DNA structure containing single HMT-derived ICL. Denaturing PAGE analysis of the reaction products. 10 nM 5′-[^32^P]-labelled XL47∙47-21*∙21 was incubated for 1 hr at 37 °C either with the 500 nM NEIL3^Cat^, NEIL3^Trun^, NEIL3^FL^ or with 20 nM Nei proteins. Lanes 1–7, no piperidine treatment; lane 1, control non-treated XL47∙47-21*∙21; lanes 2–4, as 1 but with NEIL3s; lane 5, as 1 but Nei, lane 6, 21 mer duplex containing MA; lane 7, as 6 but Nei; lanes 8–11, as 1–4 but treated with light piperidine; lane 12, 21 mer D21; lanes 13–15, 21 mer U∙A duplex treated with UDG/hot piperidine, UDG/Nfo and UDG/light piperidine, respectively. Substrate and cleavage products sizes are indicated to the right of the gel. “X” denotes substrate, “21-MA” denotes 21 mer fragment containing psoralen-derived MA, “21-mer” denotes size marker, “8 ^PA^”, “8^OH^” and “8^P^” denote 8 mer fragments containing 3′-terminal PA, OH and P, respectively. For details see Materials and Methods.

It has previously been shown that the specialized TLS DNA polymerases, such as Polκ, Polη and Polν^33–35^ can synthesize across an ICL in duplex DNA with subsequent primer extension by the REV1/Polζ complex^36–39^. Additionally, the DNA translocase FANCM can promote replication fork traverse across ICLs in DNA, which enables the stalled replication fork to restart past the ICL and continue DNA synthesis without lesion repair^22^. Based on these observations, we may hypothesize further by suggesting that DNA replication can directly bypass an ICL, without the involvement of incision steps to unhook the ICL, either on one DNA strand to generate an uninterrupted three-stranded DNA structure, or on both DNA strands to generate a four-stranded DNA structure still containing the unrepaired ICL. Here, we examined whether a four-stranded DNA structure composed of two duplex DNA molecules crosslinked to each other by HMT or 8-MOP are substrates for Nei family DNA glycosylases. For this, we constructed the four-stranded 5′-[^32^P]-labelled XL47∙47-21*∙21 and XL47∙47*−21∙21 oligonucleotides containing the 21 mer D21∙C21 oligonucleotide duplex crosslinked to the 47 mer C47∙D47 duplex (see Fig. 1, panels H and L). Incubation of XL47∙47-21*∙21, containing an HMT-derived ICL, with *E. coli* Nei generated a major 8 mer cleavage product (Fig. 4, lane 5) which migrated similar to the 8 ^P^ mer size marker containing a 3′-phosphate (lane 13) and the 8 ^P^ mer cleavage product generated by Nei when acting upon the 21 mer duplex containing MA (lane 7). Whereas, incubation of XL47∙47-21*∙21 with human NEIL3^Cat^ and piperidine generated 8 mer and 21 mer cleavage products (lane 9), the former migrated similar to the 8^PA^ mer size marker (lane 15) and the latter migrated similar to the 21 mer fragment containing MA (lane 6). Interestingly, the NEIL3-released 21 mer excision product did not disappear after piperidine treatment (lanes 9–10), but migrated similar to 21 mer fragment containing MA (lane 6) but more slowly than the 21 mer size marker (lane 12). As expected, no cleavage products were observed when the four-stranded 5′-[^32^P]-labelled XL47∙47-21*∙21 was incubated with increasing amounts of the catalytically deficient NEIL3^Cat^-K81A mutant with or without piperidine treatment (Supplementary Fig. S7). Altogether, these results suggest that the Nei-like DNA glycosylases are able to repair an ICL within a four-stranded DNA structure by excising crosslinked thymine, either in a short, labelled 21 mer D21*∙C21 duplex, or in a long non-labelled 47 mer C47∙D47 duplex.

Similar results were obtained when incubating the Nei-like DNA glycosylases with the HMT-derived four-stranded XL47∙47*−21∙21 substrate in which the 47 mer C47 oligonucleotide was 5′-[^32^P]-labelled (Fig. 1, panel L and Supplementary Fig. S8) and also with the 8-MOP-derived XL47∙47-21*∙21 (where either 21 mer C21 or D47 mer is 5′-[^32^P]-labelled) (Supplementary Fig. S9). Thus, action of Nei and NEIL1 on four-stranded DNA generates two free duplex molecules each containing a single-strand break and two thymine bases crosslinked to each other as end products of the reaction. Whereas, action of NEIL3 on four-stranded DNA generates two free duplex molecules, one containing an abasic site and the other containing a dTMP crosslinked to an extrahelical thymine base. Schematic representation of the mechanism of action of Nei-like DNA glycosylases upon four-stranded DNA structure is shown in Fig. 3B.

### Relative efficiencies of human NEIL proteins when excising an ICL and non-bulky Sp lesion

**Figure 5:**
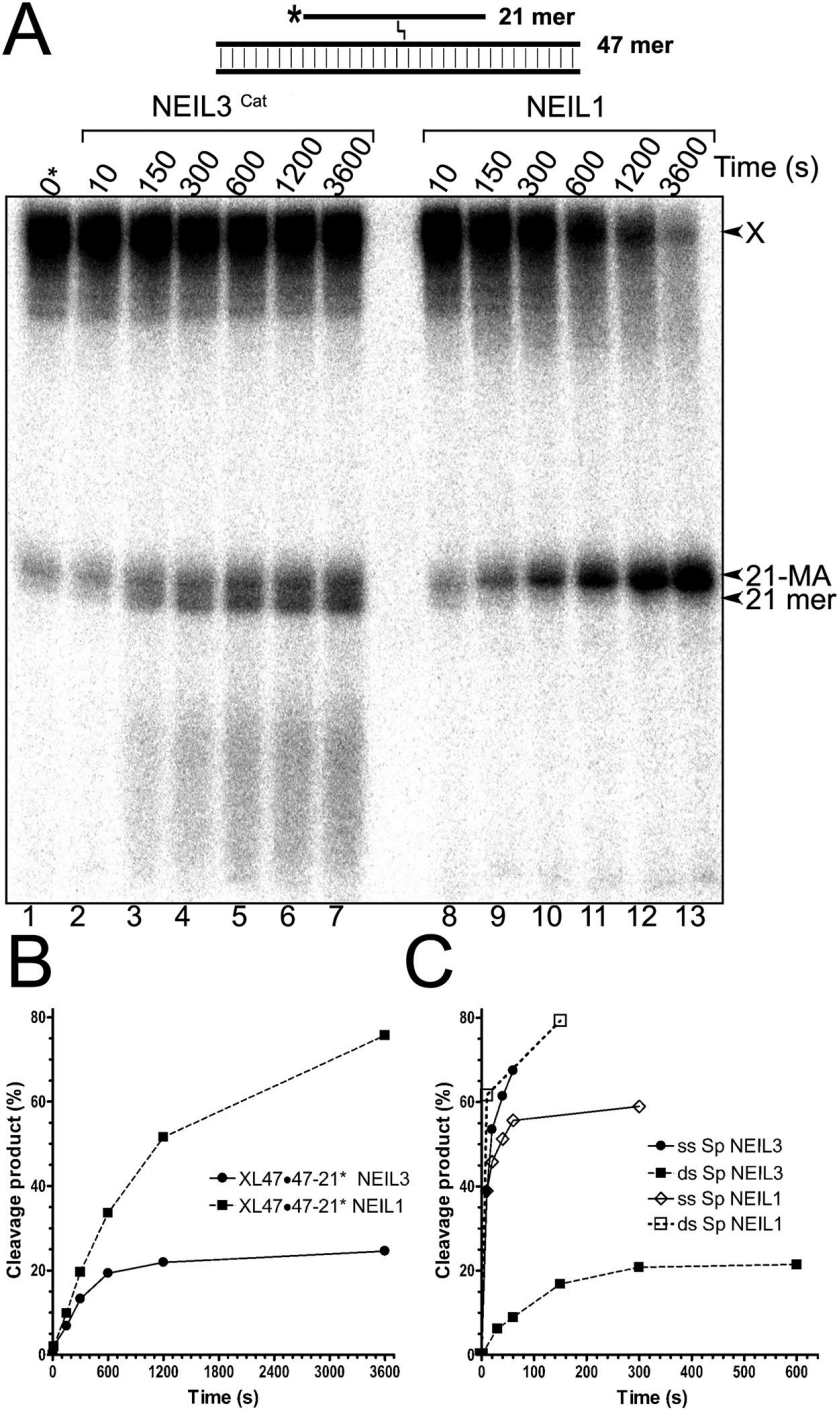
Time dependent excision of three-stranded DNA structure containing single HMT-derived ICL by NEIL3^Cat^ and NEIL1. (**A**) Denaturing PAGE analysis of the reaction products. 10 nM 5′-[^32^P]-labelled XL47∙47-21* was incubated with 300 nM NEIL3^Cat^ and 50 nM NEIL1 at 37 °C for varied periods of time up to 1 h. Reaction products were not treated with piperidine. Lane 1, control non-treated XL47∙47-21*; lanes 2–7, as 1 but with NEIL3^Cat^; lanes 8–13, as 1 but with NEIL1. Substrate and cleavage products sizes are indicated to the right of the gel. “X” denotes substrate, “21-HMT-T” denotes 21 mer excision product containing HMT crosslinked free Thymine base, “21 mer” denotes 21 mer size marker. For details see Materials and Methods. (**B**) Graphical representation of NEIL3^Cat^ and NEIL1 time kinetics on XL47∙47-21* DNA substrate. (**C**) Graphical representation of NEIL3^Cat^ and NEIL1 time kinetics on 17 mer ssDNA and dsDNA fragment containing single Sp residue.

Human NEIL1 and NEIL3 can excise a psoralen-induced ICL in three- and four-stranded DNA structures. To compare the catalytic efficiencies of these human DNA glycosylases we measured time kinetics of the excision of a 21 mer oligonucleotide in a three-stranded DNA structure and the small non-bulky spiroiminodihydantoin (Sp) lesion in 17 mer ssDNA. For this, the 5′-[^32^P]-labelled XL47∙47-21* and Sp were incubated in the presence of 300 nM NEIL3^Cat^ and 50 nM NEIL1 for increasing periods of time up to 1 hr at 37 °C. As shown in Fig. 5, both enzymes cleave the ICL in a time-dependent manner. NEIL1 exhibits linear time-dependent kinetics and cleaves ~75% of XL47∙47-21* after 1 hr of incubation (Fig. 5A, lane 13 and B), whereas NEIL3^Cat^-catalyzed excision of the 21 mer fragment reaches a plateau at ~20% of cleavage after only 10–20 min incubation (Fig. 5A, lanes 4–5 and B). These results suggest that NEIL1-catalyzed excision of an ICL in a three-stranded DNA structure is more efficient than that of NEIL3^cat^. To evaluate the DNA substrate preferences of the human enzymes, we plotted the time-dependent excision of Sp residue in ssDNA and dsDNA by NEIL3 and NEIL1, respectively. As shown in Fig. 5B,C, both human NEIL proteins strongly prefer a non-bulky modified base in ssDNA and dsDNA to a bulky ICL in three-stranded DNA. NEIL1 exhibits fast kinetics by excising more than 50% of Sp in dsDNA and ssDNA after 10 and 60 seconds incubation, respectively, whereas NEIL3^cat^ excises 60% of Sp in ssDNA after 40 sec, but only 20% Sp in dsDNA after 5 min incubation indicating strong preference of NEIL1 to dsDNA and NEIL3 to ssDNA (Fig. 5C). By comparison, NEIL1 and NEIL3^cat^ excise around 50% and 20% of the crosslinked 21 mer fragment after 20 min, respectively, thus requiring more then 100-fold longer incubation time to excise similar amount of DNA substrate as compared to Sp in dsDNA and ssDNA, respectively (Fig. 5B,C).

## Discussion

Here, using a biochemical approach we have characterized the DNA substrate specificities of human NEIL1, NEIL3 and bacterial Nei DNA glycosylases. The results revealed that the Nei-like DNA glycosylases unhook long 21 and 47 mer psoralen-crosslinked DNA fragments in three- and four-stranded DNA structures. Our data confirms that human NEIL3 acts as a monofunctional DNA glycosylase, in the presence of Sp, three- and four-stranded ICLs, leaving an abasic site as the end product and excises damaged bases preferentially from ssDNA. Under the experimental conditions used, we observed that NEIL3-catalyzed excision of ICLs does not go to completion even at the 50 fold molar excess of enzyme relative to DNA (Fig. 2B). We hypothesize that maximal activity of NEIL3, particularly *in vivo*, requires a scaffolding factor that may assemble DNA repair proteins and direct them to the lesion site. While the mechanisms of action of Nei and NEIL1 were very similar, the mechanism of unhooking of an ICL by NEIL3 was unique. For instance, in the long three-stranded DNA containing ICL DNA substrate, NEIL3 excised crosslinked thymine located either in ssDNA or in duplex DNA with approximately the same preference and generated two DNA cleavage products, one with an abasic site and another with a dTMP crosslinked to a thymine base (Figs 2 and 3A and Supplementary Figs S5, S6). In contrast, Nei and NEIL1 excised uniquely, the damaged thymine in duplex DNA and generated ssDNA fragments with a dTMP crosslinked to thymine base and duplex DNA with a single-nucleotide gap flanked with 3′ and 5′ phosphate groups of the duplex (Fig. 3A). In the four-stranded DNA structures with an ICL, all three Nei-like DNA glycosylases excised crosslinked thymine bases either in short or long duplex DNA with no apparent preference for either (Figs 3B and 4, and Supplementary Figs S8, S9). Interestingly, although NEIL3 has a strong preference for ssDNA substrates^15,16^, it was still able to unhook an ICL in the four-stranded DNA substrate which was composed of two DNA duplexes (Fig. 4 and Supplementary Fig. S8).

DNA replication of a small DNA plasmid in *Xenopus* egg extracts converts the DNA duplex surrounding the ICL into an X-shaped structure owing to replication fork convergence at the lesion site, thus offering the possibility of Nei-like enzymes to repair the ICL^28^. In our earlier work, we demonstrated that human NEIL1, contrary to NEIL3, cannot excise a crosslinked thymine base when present in ssDNA^26^. This may explain the non-involvement of NEIL1 in the unhooking of X-shaped DNA structures generated by replication fork convergence. However, NEIL1 can participate in the downstream repair steps after the initial unhooking of the ICL by NEIL3, removing the remaining crosslinked thymine base in duplex DNA. It is tempting to speculate that the capacities of Nei-like DNA glycosylases to unhook psoralen-induced ICLs in the long three- and four-stranded DNA structures indicates that these DNA substrates may also occur *in vivo*. Intriguingly, Huang and colleagues demonstrated that in mammalian cells FANCM, a DNA translocase, promotes replication fork traverse past ICL sites in DNA without lesion repair, suggesting that covalently linked DNA strands are not absolute blocks to DNA replication^22^. These authors proposed that FANCM moves the CMG helicase (a complex of Cdc45, MCM2-7, and GINS) through the ICL and restarts the replication fork downstream of the ICL by initiating, first the DNA synthesis of the leading strand and then the lagging strand synthesis. Thus, in both models the replication forks converge on an ICL and the single fork traverse of an ICL generates a similar X-shaped DNA structure around the lesion site, which may be required to initiate ICL unhooking^23^.

Based on these observations and our new biochemical data, we propose a putative model for the mechanism of ICL repair in mammalian cells that implicates the FANCM-mediated formation of long uninterrupted three- and four-stranded DNA structures after replication fork bypass of an ICL, that are then processed by NEIL1 and NEIL3 to unhook crosslinked nascent DNA strands (Supplementary Fig. S10). In this model, we hypothesize that FANCM promotes leading strand translesion synthesis through an ICL which would generate a long uninterrupted three-stranded DNA structure with an unrepaired ICL that covalently connects ssDNA with dsDNA. In a second step, after bypass of the ICL, FANCM would enable bypass of lagging strand synthesis through the ICL. After two rounds of ICL bypass, maturation of the lagging strands would generate a long uninterrupted four-stranded DNA structure (Supplementary Fig. S10). Both NEIL1 and NEIL3 would be able to unhook three- and four-stranded DNA replication intermediates and separate covalently linked sister chromatids. Yet, in the case of three-stranded DNA, the complete removal of ICL adducts in both unhooked DNA molecules would require both NEIL1 and NEIL3 DNA glycosylases. NEIL3 unhooks three-stranded DNA and generates ssDNA with an abasic site and dsDNA with crosslinked thymine base. The latter product is not a substrate for NEIL3, but NEIL1 and thus it would be removed by the latter to generate nicked duplex DNA (Fig. 3A). Whereas, when NEIL1 unhooks three-stranded DNA, it generates dsDNA with a nick and ssDNA with a crosslinked thymine base. In this case, the ssDNA adduct is not a substrate for NEIL1, but may be processed further in the TLS pathway. NEIL1, but not NEIL3, is able to completely remove an ICL in four-stranded DNA without the help of its counterpart and generate two nicked duplex molecules and two crosslinked thymine bases as the end products of the reactions (Fig. 3B). The ssDNA containing an abasic site, a NEIL3 repair product of three-stranded DNA, can be used as a template by TLS-specific DNA polymerases to generate an intact daughter chromosome. The duplex DNA with a nick flanked by 3′- and 5′-phosphates, a NEIL1 repair product of three- and four-stranded DNA, is further processed by polynucleotide kinase phosphatase or APE1 in short-patch BER^26,40^. Thus, our hypothetical model offers a relatively simple, safe and elegant way to repair ICLs during DNA replication. FANCM-mediated traverse and translesion synthesis through an ICL enables replication fork bypass and generation of uninterrupted three- and four-stranded DNA structures. These replication intermediates are unhooked by Nei-like DNA glycosylases without generation of toxic DSBs and the remaining reaction products of NEIL1 and NEIL3 are further processed by BER to generate intact daughter chromosomes. Intriguingly, this hypothetical FANCM/NEIL-dependent pathway does not require involvement of other DNA repair systems such as NER, HR, structure-specific endonucleases or the Fanconi anemia system and thus does not generate highly genotoxic DNA repair intermediates.

### Conclusion

Recent advances in the field demonstrated that ICL repair in eukaryotic cells is coupled to DNA replication and proceeds *via* two alternative pathways: (*i*) incision-dependent, which engenders replication fork collapse and generation of DSBs and, (*ii*) incision-independent, which involves unhooking by a NEIL3 DNA glycosylase. In this study, we demonstrated that human NEIL1, NEIL3 and bacterial Nei proteins can unhook psoralen-derived ICLs in three- and four-stranded DNA structures *via* hydrolysis of the glycosidic bond between the adducted base and deoxyribose sugar, thus avoiding the generation of toxic DSBs. Unusual DNA substrate specificities of the Nei-like DNA glycosylases point to the occurrence of long uninterrupted three- and four-stranded DNA structures *in vivo*, which may be generated *via* FANCM mediated replication fork bypass of an ICL. Interestingly, Nei-like DNA glycosylases can also participate in the replication-coupled, incision-dependent repair of psoralen-derived ICLs by excising a short unhooked oligomer fragment from a three-stranded DNA repair intermediate. Involvement of BER in ICL and DNA-protein cross-link repair is evolutionary conserved from *E. coli* to human cells. Appearance of Nei-like DNA glycosylases in addition to the 8-oxoguanine specific DNA glycosylases, Fpg/OGG1, in many different species during evolution imply that cells evolved a genuine repair mechanism in which complex DNA lesions can be removed in a relatively simple manner.

## Materials and Methods

### Reagents and oligonucleotides

Chemical reagents including HMT and 8-MOP were purchased from Sigma-Aldrich (France). All regular DNA oligonucleotides were purchased from Eurogentec (Seraing, Belgium). Sequences of all the oligonucleotides used in this work are described in Supplementary Table S2. The 17-mer oligonucleotide d(CCACCAACSpCTACCACC) containing single spiroiminodihydantoin (Sp) lesion at position 9 was a gift from Dr Nicolas Geacintov (New York University, NY, USA) and was synthesized as described^41^. Complementary oligonucleotides were hybridized to obtain duplexes referred to as D21∙C21, D21∙C47 and C47∙D101. Crosslinked D21-C47 and C47-D101 were hybridized with complementary D47 and C101 oligonucléotides to obtain three-stranded DNA structures XL47∙47-21 and XL101∙101-47. Crosslinked XL21-21 and XL47-21 oligonucleotide duplexes were used to generate MAs and ICLs. To obtain oligonucleotide containing single MAp residue, the denaturing gel purified crosslinked DNA duplexes were treated with hot alkali as described^29^. Chemical structures of psoralen-DNA adducts used in this work are shown in Supplementary Table S1.

### Expression and purification of DNA glycosylases

The purified BER proteins including human NEIL1, *E. coli* uracil-DNA glycosylase (UDG), endonuclease IV (Nfo) and Nei were from laboratory stock. The activities of various DNA repair proteins were tested using their principal substrates and were checked just prior to use. The bacterial expression vector phNEIL1 was generously provided by Drs Hiroshi Ide (Hiroshima University, Japan)^42^. The full length native NEIL1 was purified as described previously^42^. The expression vector pET13a-Nei for the *E. coli* Nei protein was generously provided by Dmitry Zharkov (ICBFM, Novosibirsk, Russia)^43^. The *E. coli* Nei protein was purified as described previously^44^.

Purification of human NEIL3 proteins. As the N-terminal methionine residue adjacent to catalytic valine of NEIL3 is poorly removed during protein maturation in host bacterial cells, Wallace and colleagues developed a protocol in which they co-expressed a modified *E. coli* methionine aminopeptidase (EcoMap, Y168A) with mammalian NEIL3 proteins to increase the amount of active DNA glycosylase in the preparation^45^. Expression and purification of recombinant hNEIL3 proteins was achieved by the molecular cloning of hNEIL3^Cat^, ^Trun^ and ^FL^ cDNA sequences into the pETDuet2-*EcoMap*-ORF6- bicistronic expression vector (a generous gift from S.S. Wallace to J.L. Parsons). Full details of the cloning, expression and purification will be reported elsewhere. Vectors were transformed into Rosetta 2 (DE3) expression hosts for recombinant protein expression and subsequent FPLC purification following similar methods to those described by Liu and colleagues^45^.

To prepare the hNEIL3^Cat^-K81A-His expression vector, the pETDuet2-*EcoMap-*ORF6-hNEIL3^Cat^ expression vector was subjected to site directed mutagenesis using 1.75 units of Pfu Ultra DNA polymerase (Agilent technologies). PCR primers were used, to mutate the codon encoding for a lysine residue at amino acid position 81 to an alanine, to remove glycosylase and AP lyase activity as described by Krokeide and colleagues^15^. Products were digested with 10 units of DpnI restriction endonuclease and transformed into Nova XG cloning hosts by electroporation and confirmed by Sanger sequencing as previously described. Expression and purification of hNEIL3^Cat^-K81A was carried out following the same methods used for purification of hNEIL3^Cat^.

### Preparation of psoralen ICLs

30 pmol of 5ʹ-[^32^P]-labelled D21 or C47 oligonucleotide were mixed with 120 pmol of non-labeled D21 or C47 oligonucleotide, respectively. 130 pmol of total D21 or C47 oligonucleotide was mixed with 143 pmol of C47 and D47 or D101 respectively and annealed, generating 47∙21*, 47*∙21 or 101∙47* oligonucleotide duplexes. 117 pmol of total oligonucleotide from the annealing reactions was incubated with 0.5 µM HMT or 8-MOP for 15 min in the dark at room temperature in dH_2_O at a final volume of 100 µl. Samples were then irradiated at 365 nm and 240 kJ/m2 for 30 min (HMT) or 60 min (8-MOP) on ice. Crosslinked oligonucleotides were then separated by 20% denaturing-PAGE and DNA bands containing psoralen crosslinked oligonucleotides were excised. It should be noted that photoreaction of asymmetric psoralens with the DNA duplex yields two orientational isomers of the ICL that migrate in denaturing PAGE as two bands^29,46^. Crosslinked oligonucleotides were eluted from gel strips in dH_2_O and precipitated in 2% LiClO_4_/acetone. Eluted crosslinked oligonucleotides were reconstituted in dH_2_O for use in further annealing reactions, oligonucleotide substrate analysis and DNA glycosylase activity assays. Preparation of all psoralen adducted oligonucleotides were conducted in the same manner.

To prepare three- and four-stranded crosslinked DNA oligonucleotides containing psoralen-derived ICLs, the XL47-21 and XL101-47 duplexes were annealed with complementary 21, 47 and 101 mer oligonucleotides, respectively.

### DNA repair assay

The standard reaction mixture (20 µL) for ICL-specific DNA glycosylase activity contained 10 nM of 5´-[^32^P]-labelled DNA substrate, incubated with reaction specific concentrations of enzyme, diluted in RDWB buffer (25 mM HEPES-KOH pH 7.6, 100 mM KCl, 1 mM EDTA, 1 mM DTT and 17% glycerol) and DNA glycosylase reaction buffer (25 mM HEPES-KOH pH 7.6, 100 mM KCl, 1 mM EDTA, 1 mM DTT and 100 µg/ml BSA) for 60 min at 37 °C. Reactions were stopped by the addition of 0.2% SDS and 4 mM EDTA and incubated if necessary for a further 30 min at 37 °C in the presence of 10% piperidine, to cleave AP sites by β-elimination. The reaction was then stopped with the addition of 20 mM EDTA and neutralised with 40 mM HCl. The samples were desalted by passing through house-made spin-down columns filled with Sephadex G25 (GE Healthcare) equilibrated in 7.5 M urea. Desalted reaction products were separated by electrophoresis in denaturing 20% (w/v) polyacrylamide gel (7.5 M urea, 0.5 × TBE), gels were exposed to a Fuji FLA-9500 Phosphor Screen and analyzed using Image Gauge V3.12 software.

### Preparation of DNA size markers containing 3′-terminal modifications

The DNA fragments containing 3′-terminal phopshate (P), hydroxyl (OH) and α,β-unsaturated phosphoaldehyde (PA) groups were prepared by the cleavage of duplex oligonucleotide containing a single AP site with hot piperidine, *E. coli* Nfo and light piperidine, respectively. For this 5ʹ-[^32^P]-labelled D21-U and C47-U oligonucleotides containing a single Uracil residue at position 9 and 24, respectively, were hybridized with complementary regular C21 and D47 oligonucleotides to obtain 21 and 47 mer duplexes with single U∙A base pair. The U∙A duplexes were incubated in the presence of 20 nM *E. coli* UDG in DNA glycosylase reaction buffer for 30 min at 37 °C to obtain AP sites. Then 21 and 47 mer duplexes with an abasic site were incubated for 30 min at 37 °C in the presence of 10% piperidine to cleave AP sites *via* β-limination and produce 5ʹ-[^32^P]-labelled 8 and 23 mer cleavage fragments containing 3′-terminal PA group. Treatment by hot piperidine (30 min at 90 °C in the presence of 10% piperidine) incised AP sites in DNA *via* β,δ-elimination and produced 5ʹ-[^32^P]-labelled 8 and 23 mer cleavage fragments containing 3′-terminal P group. Hydrolytic cleavage of AP sites in DNA by bacterial AP endonuclease Nfo produced 5ʹ-[^32^P]-labelled 8 and 23 mer cleavage fragments containing 3′-terminal OH group. Structures of DNA fragments with 3′-terminal modifications are shown in Supplementary Table S1.

In addition, we incubated 5ʹ-[^32^P]-labelled 21 mer D21*∙C21 and 47 mer C47*∙D47 duplexes containing a single MAp adduct with Nei to generate 8 and 23 mer cleavage fragments containing 3′-terminal P group.

### Equipment and settings

The acquisition of images of denaturing PAGE and non-denaturing PAGE gels was facilitated by exposure to a Storage Phosphor Screen BAS (GE Healthcare Life Sciences) overnight. Phosphor Screens were then imaged using the Typhoon^TM^ FLA 9500 Laser Scanner (GE Healthcare Life Sciences) with an pixel resolution of 100 µM. Retrieved images in TIFF format were then processed in Adobe photoshop CS6 software, to adjust brightness and contrast settings for appropriate visualisation of 5′-^32^P labelled DNA. Consistent adjustment of brightness and contrast was used across the entirety of the gel image. Quantification of DNA cleavage was facilitated by use of the FUJIFILM Science Lab Image Gauge Ver. 4.0 software (Fujifilm), with quantification adjusted by removal of standardised background values on each gel image. GraphPad Prism 7 software was used for generation of graphical representations of percentage cleavage quantifications. Schema were generated with use of Adobe photoshop CS6. SDS-PAGE image showing final purification products was imaged at 700 nm wavelength using the Licor Odyssey Infared imaging system.

### Data availability statement

All data generated or analysed during this study are included in this published article (and its Supplementary Information files).

## Electronic supplementary material

Supplementary Information
