# Supplementary material for: The Human DNA glycosylases NEIL1 and NEIL3 Excise Psoralen-Induced DNA-DNA Cross-Links in a Four-Stranded DNA Structure
Source: Sci Rep. 2017 Dec 12;7:17438. doi: 10.1038/s41598-017-17693-4 (PMC5727206; doi:10.1038/s41598-017-17693-4)
Supplement: Supplementary file 1 — Supplementary Information [file 41598_2017_17693_MOESM1_ESM.pdf]

Supporting Information

The Human DNA glycosylases NEIL1 and NEIL3 Excise Psoralen-Induced DNA-DNA Cross-Links in a Four-Stranded DNA Structure

Peter R. Martin[1], Sophie Couvé[2], Caroline Zutterling[3], Mustafa S. Albelazi[1], Regina Groisman[3], Bakhyt T. Matkarimov[4], Jason L. Parsons[5], Rhoderick H. Elder[1,] and Murat K. Saparbaev[3,]**

[1] Biomedical Research Centre, Cockcroft Building, University of Salford, Salford M5 4NT, UK.
[2] Ecole Pratique des Hautes Etudes, Paris, France Laboratoire de Génétique Oncologique EPHE, INSERM U753, Villejuif, France Faculté de Médecine Université Paris-Sud, Le Kremlin-Bicêtre, France.
[3] Groupe «Réparation de l'ADN», Equipe Labellisée par la Ligue Nationale Contre le Cancer, CNRS UMR8200, Université Paris-Sud, Gustave Roussy Cancer Campus, F-94805 Villejuif Cedex, France.
[4] National laboratory Astana, Nazarbayev University, Astana 010000, Kazakhstan.
[5] Cancer Research Centre, Department of Molecular and Clinical Cancer Medicine, University of Liverpool, 200 London Road, Liverpool L3 9TA, UK.

**Correspondence and request for materials should be addressed to M.K.S. and R.H.E.: phone: 33142115404; Email: smurat@igr.fr and phone: 441612953094; email: r.h.elder@salford.ac.uk*

Supplementary Tables S1-S2.

Supplementary Table S1. The skeletal structural formula of HMT generated ICL, MAp and MAf and also excision DNA products generated by Nei-like DNA glycosylases, Nfo and piperidine treatments.

Cleavage product	Chemical structure
**D21-C47 ICL**	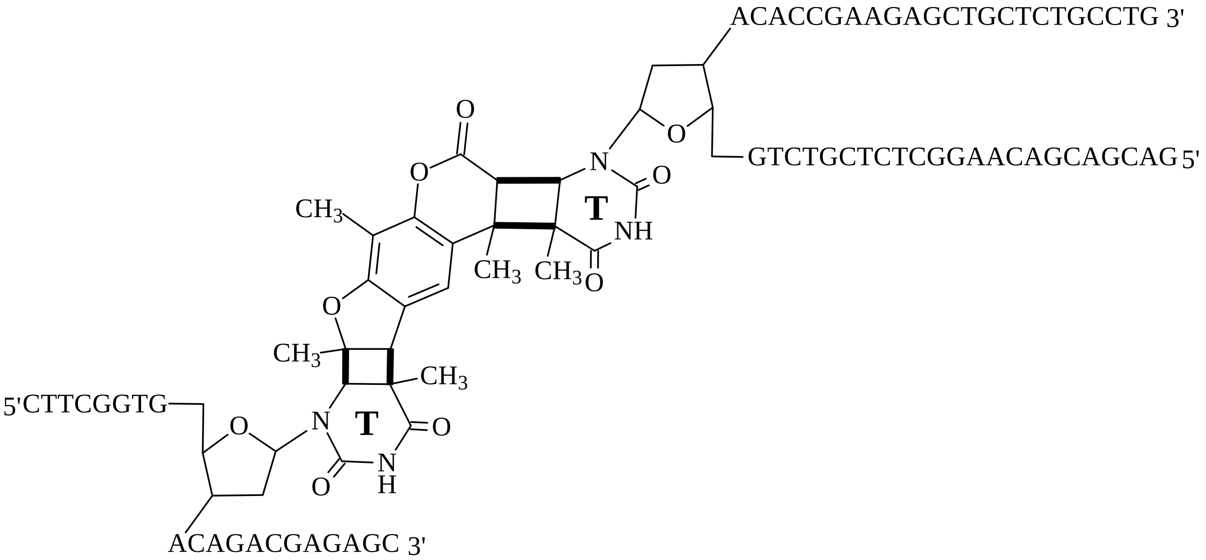
**21 mer-MAf (furan)**	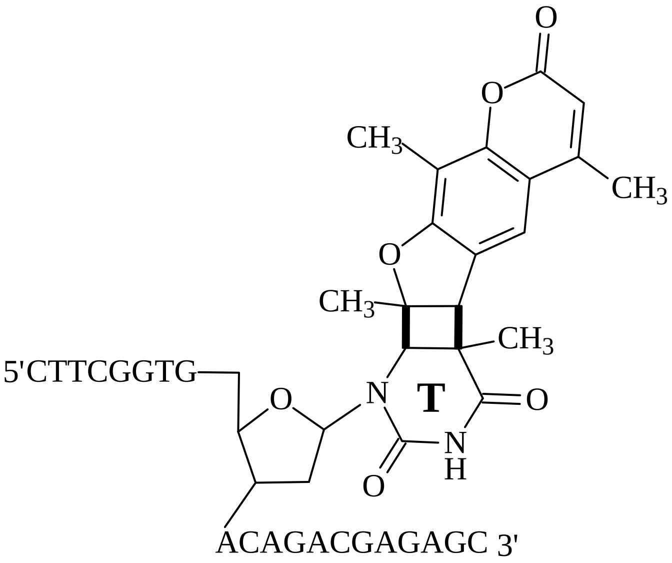

<table>
<tr><td>21 mer-MAp (pyrone)</td><td>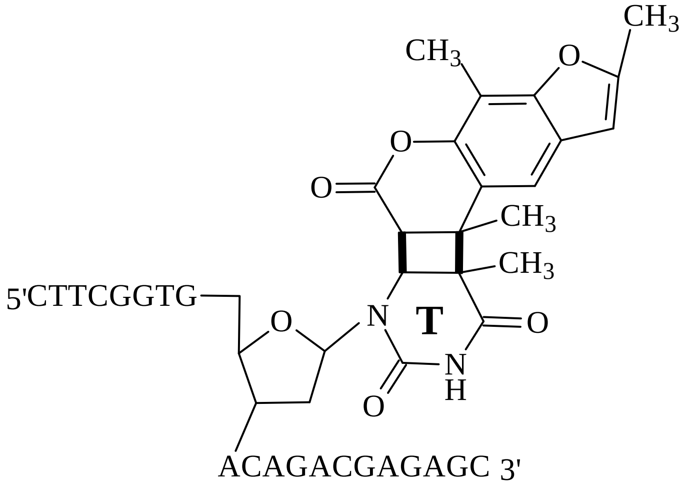
</td></tr>
<tr><td>21mer DNA(T)-HMT-T</td><td>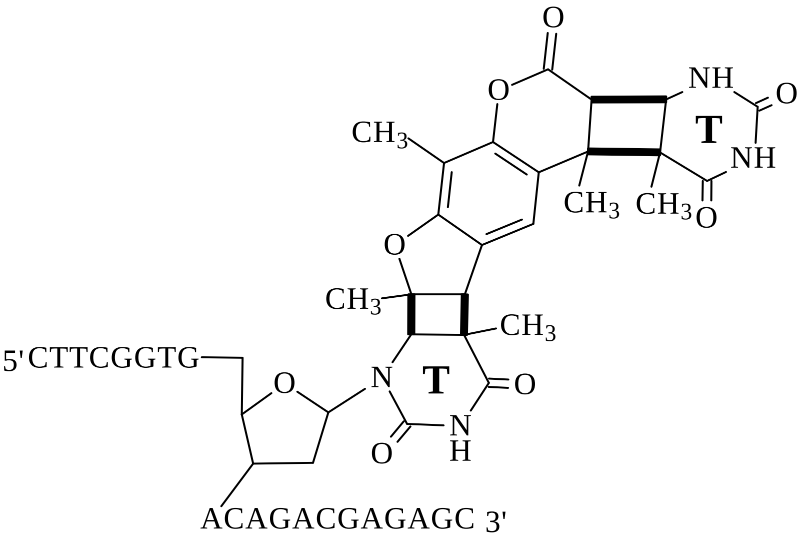
</td></tr>
<tr><td>T-HMT-T</td><td>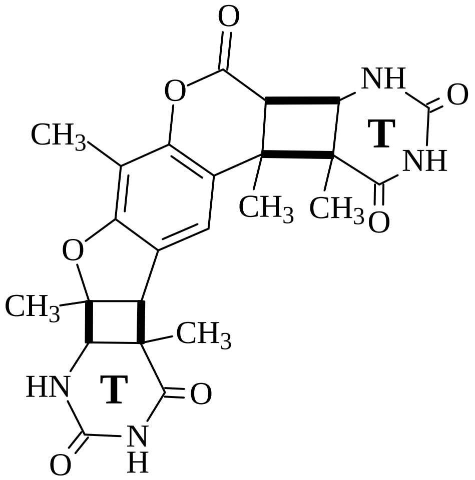
</td></tr>
<tr><td>8^{OH} mer (1.D21 2.Sp1)</td><td>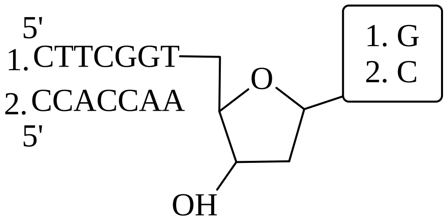
</td></tr>
</table>

**8^{PA} mer (1.D21 2.Sp1)**	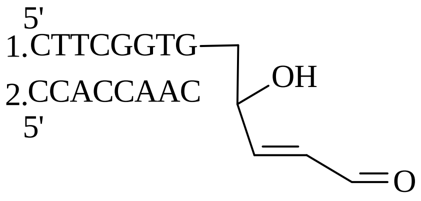
**8^{P} mer (1.D21 2.Sp1)**	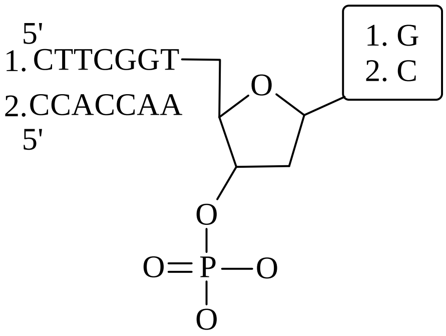

Supplementary Table S2. Sequences of oligonucleotides containing single TpA dinucleotide to generate psoralen-derived MAs and ICL and base modification.

**Oligonucleotide name**	**Oligonucleotide sequence (5'-3')**	**Position of TpA site and DNA modifications and cleavage product details**
**D21**, 21 mer	d(CTTCGGTG**TA**CAGACGAGAGC)	TpA position 9-10; excision of T9 generates 8 mer cleavage product
**C21**, 21 mer	d(GCTCTCGTCTG**TA**CACCGAAG)	TpA position 12-13; excision of T12 generates 11 mer cleavage product
**C47**, 47 mer	d(GACGACGACAAGGCTCTCGTCTG**TA**CACCGAAGAGCTGCTCTGCCTG)	TpA position 24-25; excision of T24 generates 23 mer cleavage product
**D47**, 47 mer	d(CAGGCAGAGCAGCTCTTCGGTG**T**ACAGACGAGAGCCTTGTCGTCGTC)	TpA position 23-24; excision of T23 generates 22 mer cleavage product
**C101**, 101 mer	d(GCCGGTAAAAATATACGATGGCTGCAAGACGACGACAAG*GCTCTCGTCTG**TA**CACCGAAG*AGCTGCTCTGCCTGGAGACAGCACAGCTGCCCAAGGTGTTC)	TpA position 51-52; excision of T51 generates 50 mer cleavage product
**D101**, 101 mer	d(GAACACCTTGGGCAGCTGTGCTGTCTCCAGGCAGAGCAGCT*CTTCGGTG**TA**CAGACGAGAGC*CTTGTCGTCGTCTTGCAGCCATCGTATATTTTTACCGGC)	TpA position 50-51; excision of T50 generates 49 mer cleavage product
**Sp1**, 17 mer	d(CCACCAAC[Sp1]CTACCACC)	Sp1 position 9; excision of Sp19 generates 8 mer cleavage product
**Sp1 complement**, 17 mer	d(GGTGGTAGCGTTGGTGG)	Cytosine complementary to Sp1 at position 10
**D21-U**, 21 mer	d(CTTCGGTG**U**ACAGACGAGAGC)	U position 9; excision of U9 generates 8 mer cleavage product
**C47-U**, 21 mer	d(GACGACGACAAGGCTCTCGTCTG**U**ACACCGAAGAGCTGCTCTGCCTG)	U position 24; excision of U24 generates 23 mer cleavage product

Supplemetary Figures S1-S10

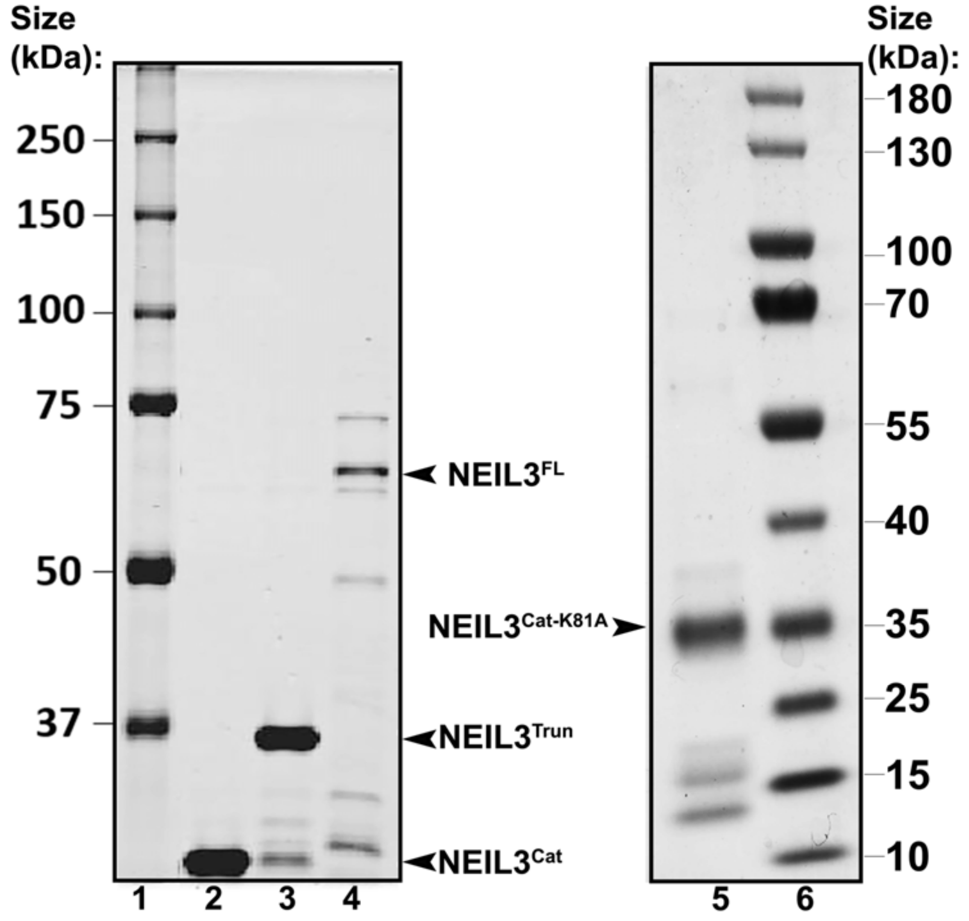

Supplementary Figure S1. SDS-PAGE analysis of the purified recombinant human NEIL3 proteins. Lane 1, molecular size markers (All Blue Precision Plus Prestained Protein Standard, BioRad); lane 2, 1 µg NEIL3^{Cat}; lane 3, 1 µg NEIL3^{Trun}; lane 4, 0.5 µg NEIL3^{FL}, lane 5, NEIL3$^{Cat-K81A}$, lane 6 molecular size markers (Page Ruler Prestained Protein Ladder, Thermo fisher). For details, see Materials and Methods.

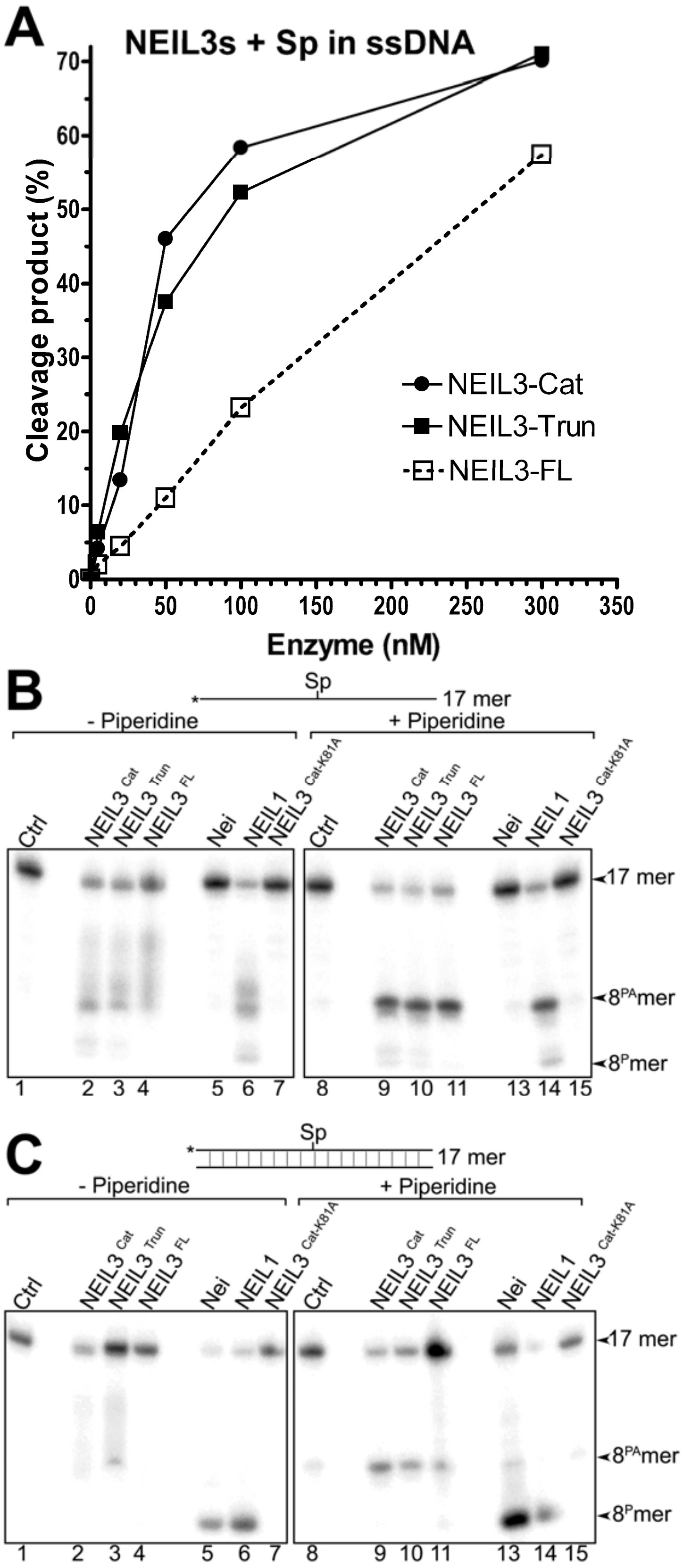

Supplementary Figure S2. **Action of Nei-like DNA glycosylases on DNA oligonucleotides containing single spiroiminodihydantoin (Sp) residue**. 10 nM 5'-[^{32}P]-labelled 17-mer Sp and Sp•C were incubated with 500 nM NEIL3^{Cat}, NEIL3^{Trun}, NEIL3^{FL} and mutant NEIL3^{Cat}-K81A proteins and 20 nM Nei and NEIL1 proteins for 1 hr at 37°C. Products of the reaction were treated or not with light piperidine and analyzed on denaturing gel to measure cleavage efficiency. (**A**) Graphical representation of the NEIL3^{Cat}, NEIL3^{Trun} and NEIL3^{FL} protein activities upon single-strand oligonucleotide containing single Sp residue. (**B**) Denaturing PAGE analysis of the action of Nei-like DNA glycosylases on single-stranded oligonucleotide containing spiroiminodihydantoin (Sp) residue. Lanes 1-7, no piperidine treatment; lane 1, control Sp; lanes 2-4, as 1 but with NEIL3^{Cat}; NEIL3^{Trun} and NEIL3^{FL}, respectively; lanes 5-7, as 1 but with Nei, NEIL1 and NEIL3^{Cat}-K81A, respectively; lanes 8-15, as 1-7 but with piperidine treatment. (**C**) Denaturing PAGE analysis of the action of Nei-like DNA glycosylases on duplex oligonucleotide containing spiroiminodihydantoin (Sp) residue. Lanes 1-7, no piperidine treatment; lane 1, control Sp•C; lanes 2-4, as 1 but with NEIL3^{Cat}; NEIL3^{Trun} and NEIL3^{FL}, respectively; lanes 5-7, as 1 but with Nei, NEIL1 and NEIL3^{Cat}-K81A, respectively; lanes 8-15, as 1-7 but with piperidine treatment. Substrate and cleavage products sizes are indicated to the right of the gel. "17 mer" denotes substrate, "8^{PA}" and "8^{P}" denote 8 mer cleavage fragments containing 3'-terminal PA and P, respectively. For details see Materials and Methods..

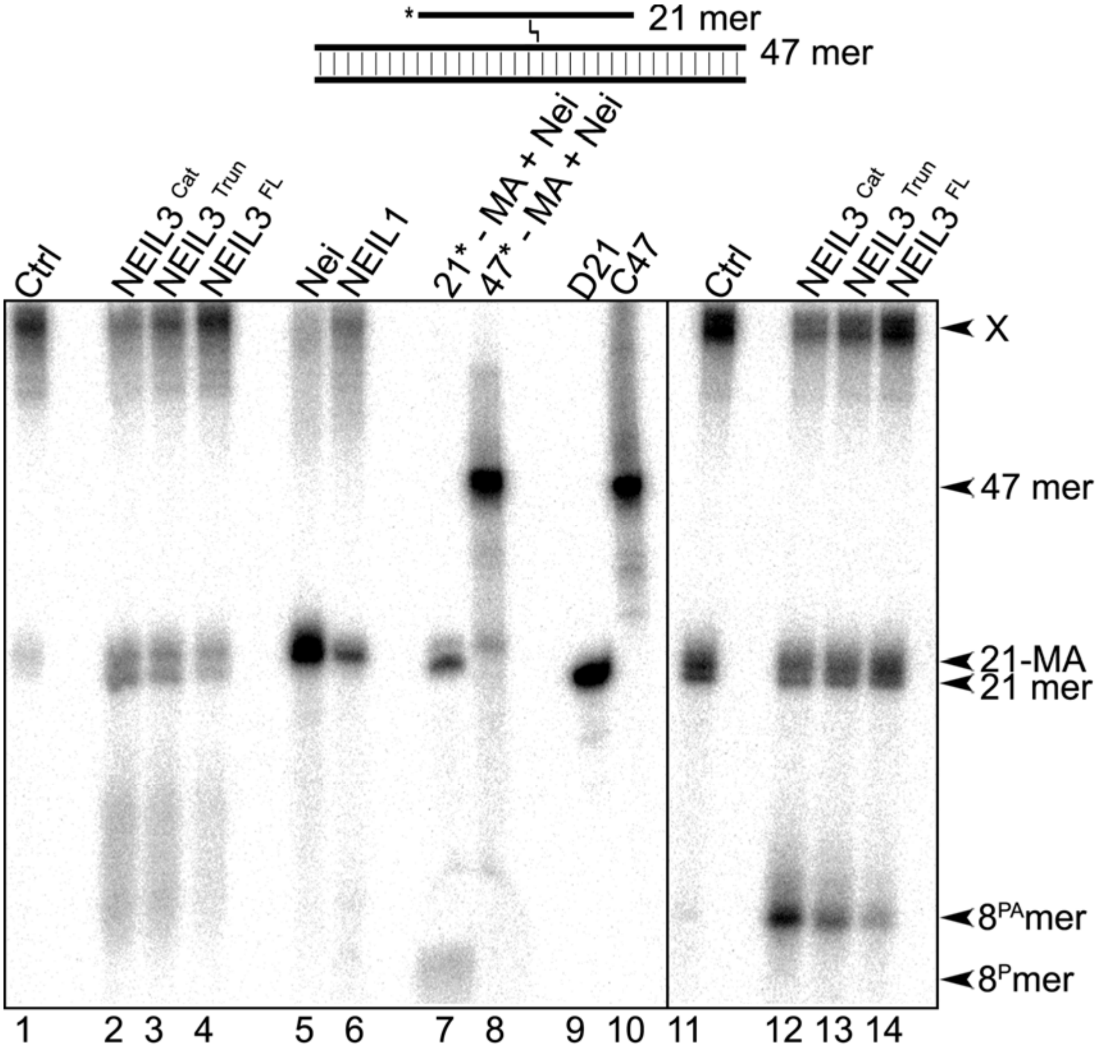

Supplementary Figure S3. **Action of Nei-like DNA glycosylases upon three-stranded DNA structure containing single 8-MOP-derived ICL**. Denaturing PAGE analysis of the reaction products. 10 nM 5'-[^{32}P]-labelled XL47•47-21* was incubated for 1 hr at 37°C either with the 500 nM NEIL3^{Cat}, NEIL3^{Trun}, NEIL3^{FL} or with 20 nM Nei and 50 nM NEIL1 proteins. Lane 1, control non-treated XL47•47-21*; lanes 2-4, as 1 but with NEIL3s; lanes 5 and 6, as 1 but with Nei and NEIL1, respectively; lanes 7 and 8, 21- and 47-mer oligonucleotides containing MA incubated with Nei; lanes 9 and 10, 21- and 47-mer size markers; lanes 11-14, as 1-4 but treated with piperidine. Substrate and cleavage products sizes are indicated to the right of the gel. "X" denotes ICL substrate, "47 mer " denotes size marker; "21-MA" denotes 21 mer fragment containing HMT-derived MA, "21 mer" 21 mer size marker, "8^{PA}" and "8^{P}" denote 8 mer cleavage fragments containing 3'-terminal PA and P, respectively. For details see Materials and Methods.

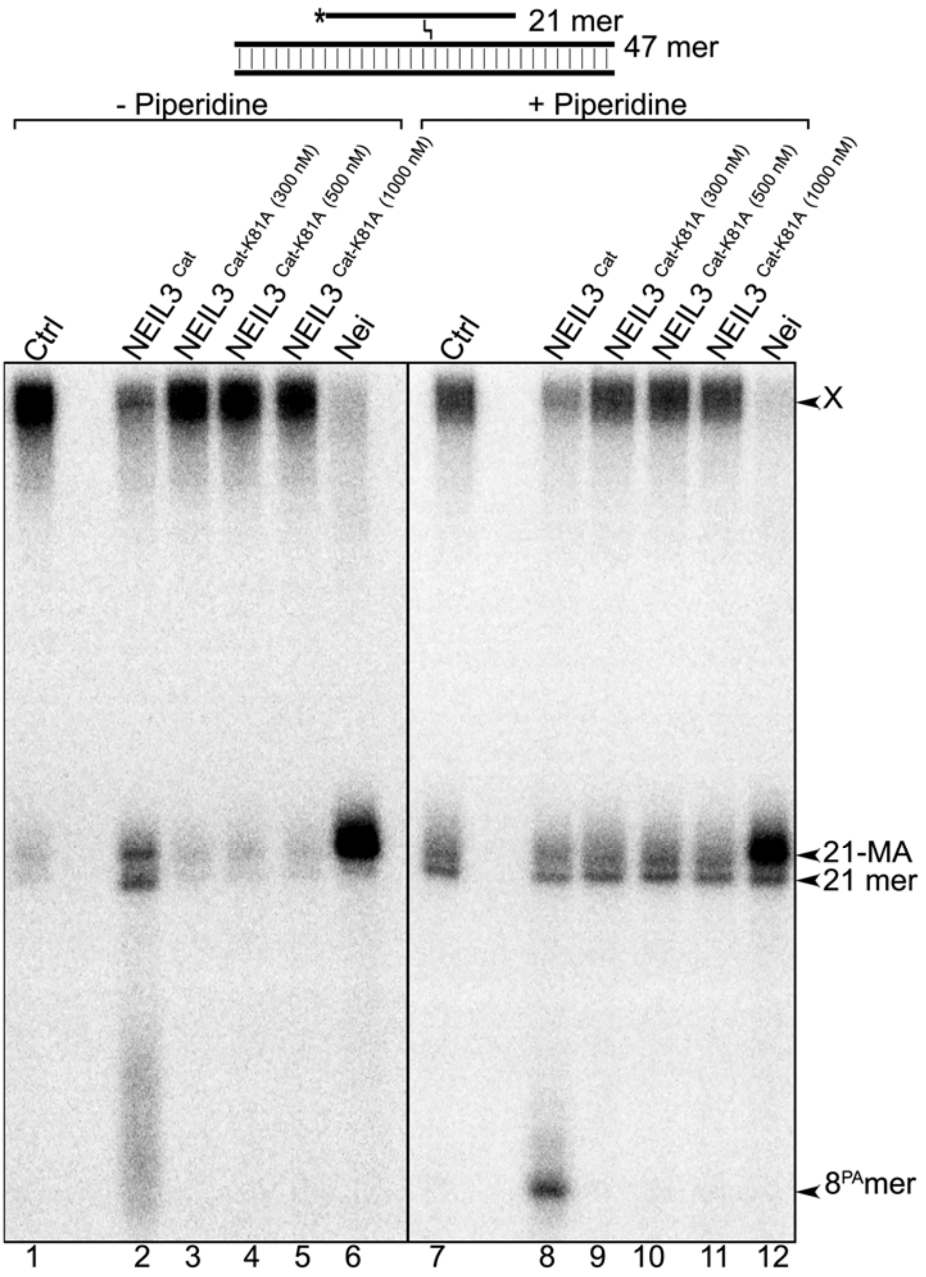

Supplementary Figure S4. **Action of NEIL3^{Cat}, NEIL3^{Cat}-K81A and Nei upon three-stranded DNA structure containing single HMT-derived ICL**. Denaturing PAGE analysis of the reaction products. 10 nM 5'-[^{32}P]-labelled XL47•47-21* was incubated for 1 hr at 37°C with the 500 nM NEIL3^{Cat}, varying concentrations of NEIL3^{Cat}-K81A and 20 nM Nei proteins. Lanes 1-6, no piperidine treatment; lane 1, control non-treated XL47•47-21*; lanes 2, as 1 but with NEIL3^{Cat}; lanes 3-5 as 1 but with 300, 500 and 1000 nM NEIL3^{Cat}-K81A, respectively; lane 6, as 1 but with Nei; lanes 7-12, as 1-6 but treated with piperidine. Substrate and cleavage products sizes are indicated to the right of the gel. "X" denotes ICL substrate, "21-MA" denotes 21 mer fragment containing HMT-derived MA, "21 mer" 21 mer size marker, "8^{PA}" denotes 8 mer cleavage fragments containing 3'-terminal PA. For details see Materials and Methods.

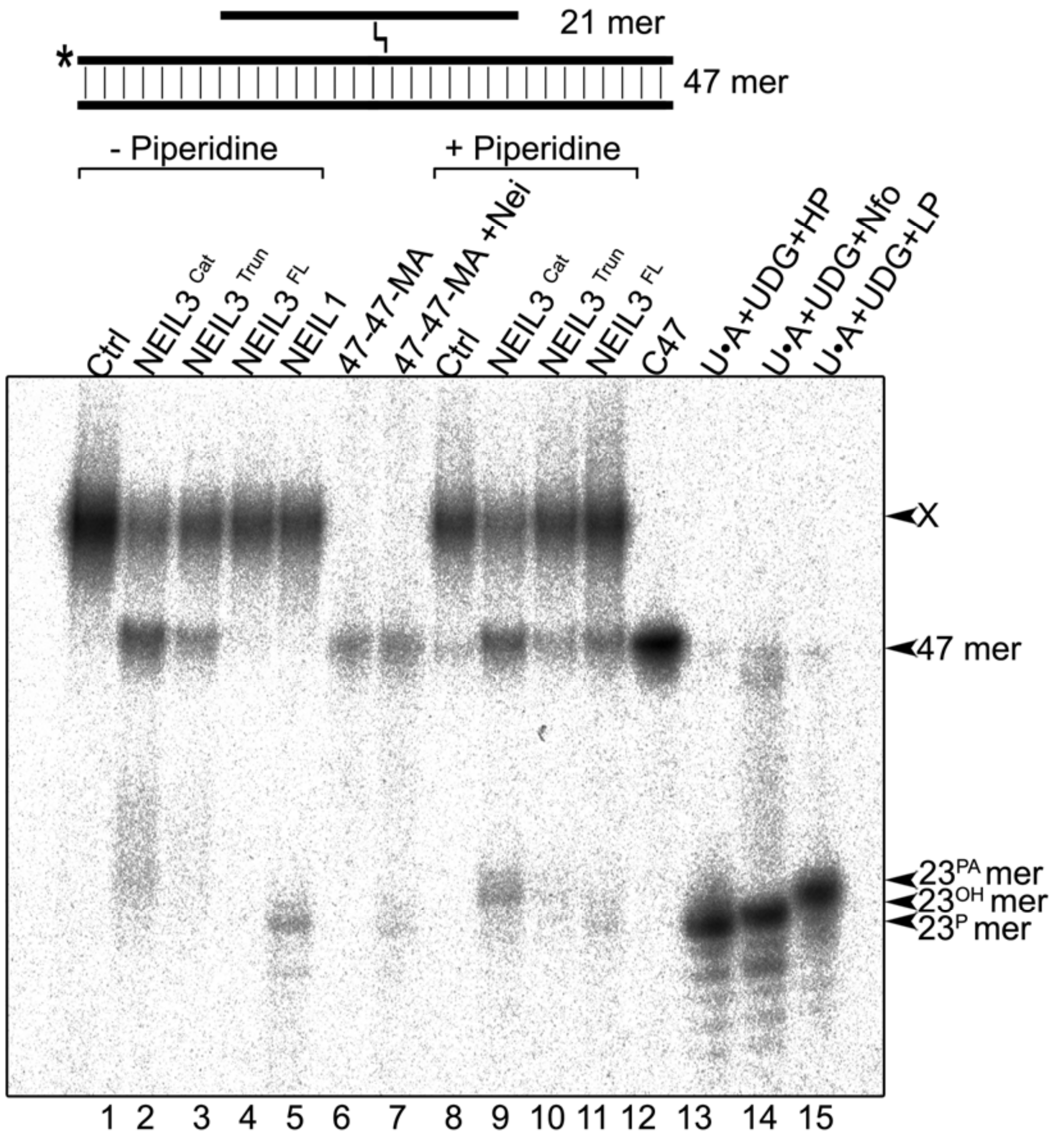

Supplementary Figure S5. **Action of Nei-like DNA glycosylases upon three-stranded DNA structure containing single HMT-derived ICL**. Denaturing PAGE analysis of the reaction products. 10 nM 5'-[^{32}P]-labelled XL47•47*-21 was incubated for 1 hr at 37°C either with the 500 nM NEIL3^{Cat}, NEIL3^{Trun}, NEIL3^{FL} or with 50 nM NEIL1 proteins. Lanes 1-7, no piperidine treatment; lane 1, control non-treated XL47•47*-21; lanes 2-4, as 1 but with NEIL3s; lane, 5 NEIL1; lane 6, 47 mer duplex containing HMT-derived MA; lane 7, as 6 but with Nei; lanes 8-11, as 1-4 but treated with light piperidine; lane 12, 47 mer size marker, C47; lanes 13-15, 47 mer U•A duplex treated with UDG/hot piperidine, UDG/Nfo and UDG/light piperidine, respectively. Substrate and cleavage products sizes are indicated to the right of the gel. "X" denotes ICL substrate, "47 mer" denotes 47 mer size marker, "23^{PA}", "23^{OH}" and "23^{P}" denote 23 mer fragments containing 3'-terminal PA, OH and P, respectively. For details see Materials and Methods.

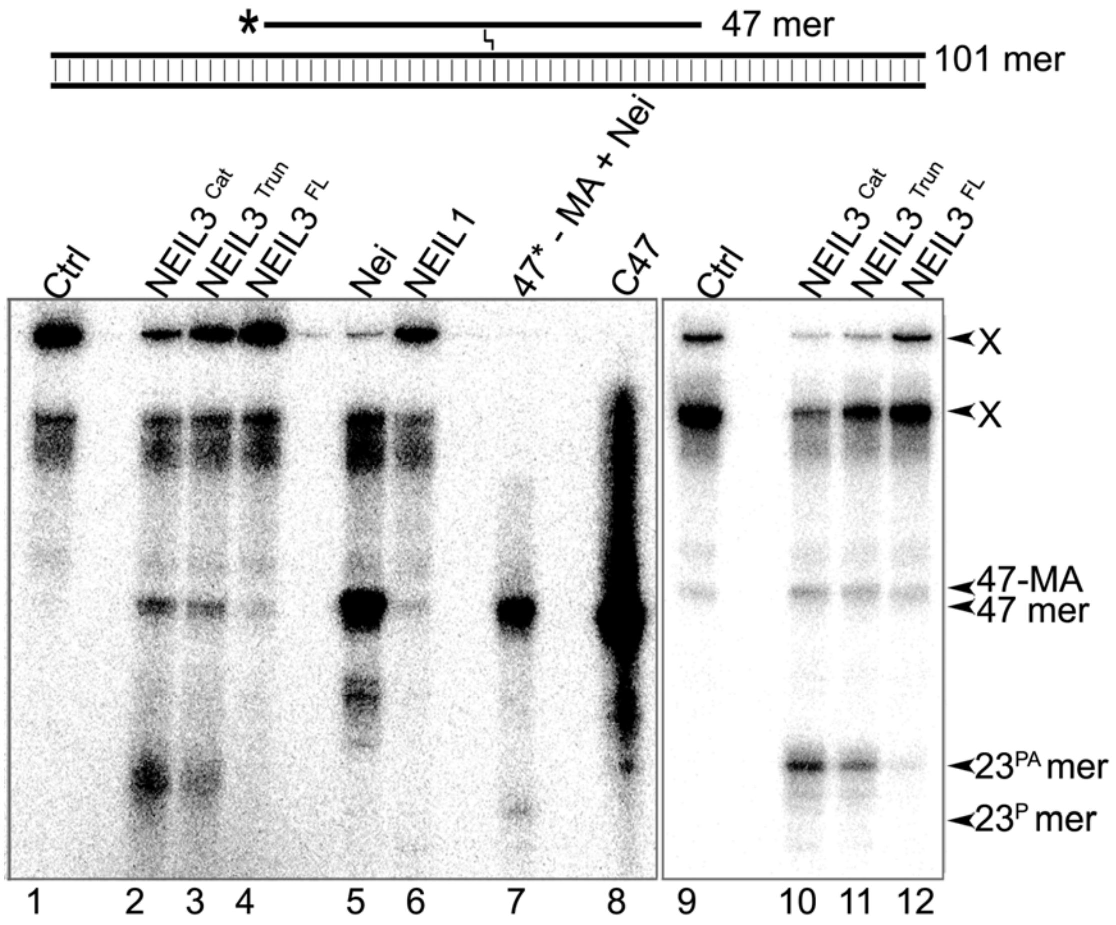

Supplementary Figure S6. **Action of Nei-like DNA glycosylases upon long three-stranded DNA structure containing single HMT-derived ICL**. 10 nM 5'-[^{32}P]-labelled XL101•101-47* was incubated with 500 nM NEIL3^{Cat}, NEIL3^{Trun} and NEIL3^{FL} proteins and 20 nM Nei and 50 nM NEIL1 proteins for 1 hr at 37°C. The products of reaction were analyzed on denaturing PAGE. Lanes 1-8, no piperidine treatment; lane 1, control non-treated XL101•101-47*; lanes 2-3, as 1 but with NEIL3s; lanes 5 and 6, as 1 but with Nei and NEIL, respectively; lane 7, 47-mer oligonucleotide containing MA incubated with Nei; lane 8, 47-mer size marker; lanes 9-12, as 1-4 but treated with piperidine. Substrate and cleavage products sizes are indicated to the right of the gel. X" denotes ICL substrate, "47-MA" denotes 47 mer fragment containing HMT-derived MA, "47 mer" denotes size marker, "23^{PA}" and "23^{P}" denote 23 mer fragments containing 3'-terminal PA and P, respectively. For details see Materials and Methods.

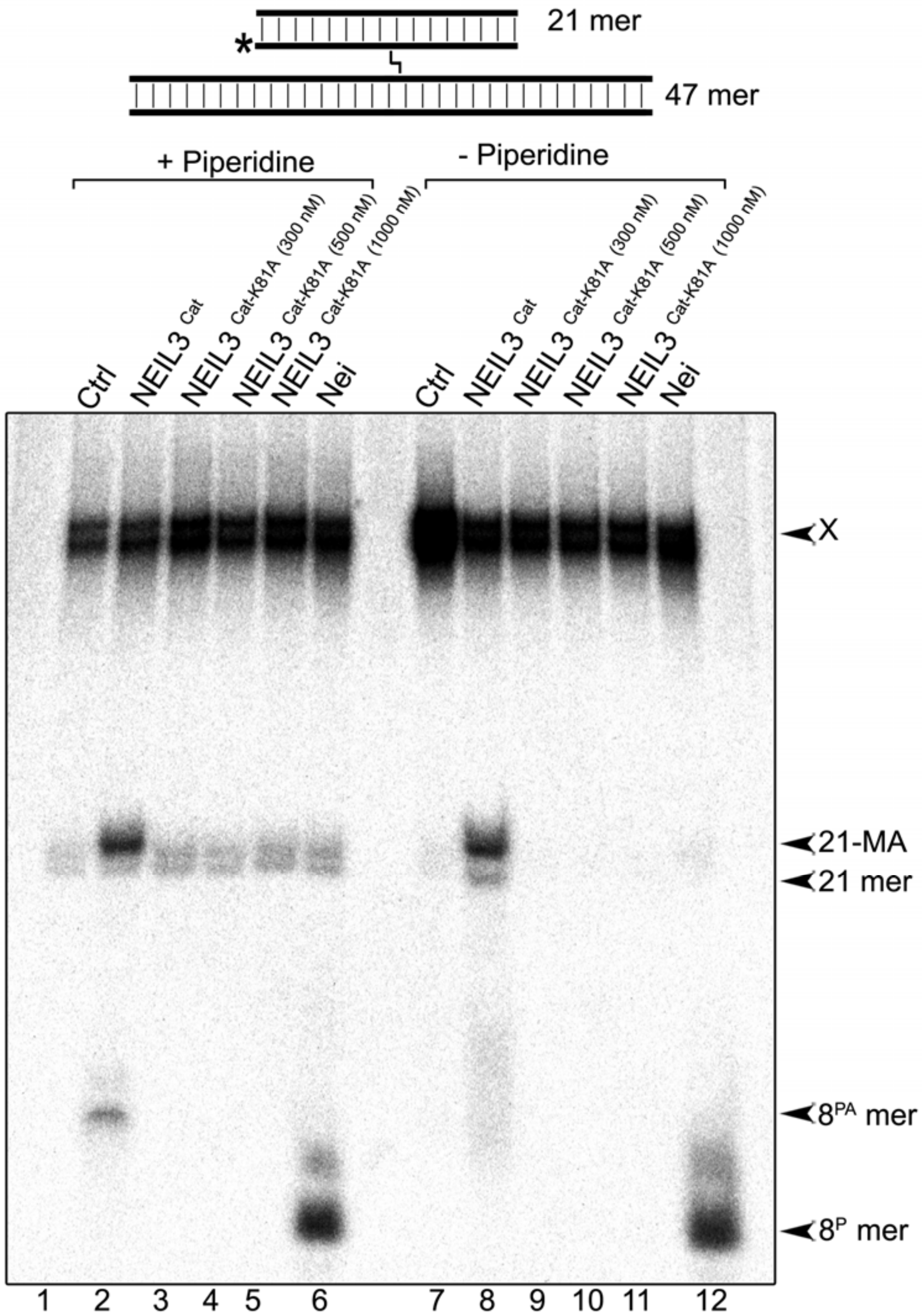

Supplementary Figure S7. **Action of NEIL3^{Cat}, NEIL3^{Cat}-K81A and Nei upon four-stranded DNA structure containing single HMT-derived ICL**. Denaturing PAGE analysis of the reaction products. 10 nM 5'-[^{32}P]-labelled XL47•47-21*•21 was incubated for 1 hr at 37°C with the 500 nM NEIL3^{Cat}, varying concentrations of NEIL3^{Cat}-K81A and 20 nM Nei proteins. Lanes 1-6, with piperidine treatment; lane 1, control XL47•47-21*; lanes 2, as 1 but with NEIL3^{Cat}; lanes 3-5 as 1 but with 300, 500 and 1000 nM NEIL3^{Cat}-K81A, respectively; lane 6, as 1 but with Nei; lanes 7-12, as 1-6 but no piperidine treatment. Substrate and cleavage products sizes are indicated to the right of the gel. "X" denotes ICL substrate, "21-MA" denotes 21 mer fragment containing HMT-derived MA, "21 mer" denotes 21 mer size marker, "8^{PA}" and "8^{P}" denote 8 mer cleavage fragments containing 3'-terminal PA and P, respectively. For details see Materials and Methods.

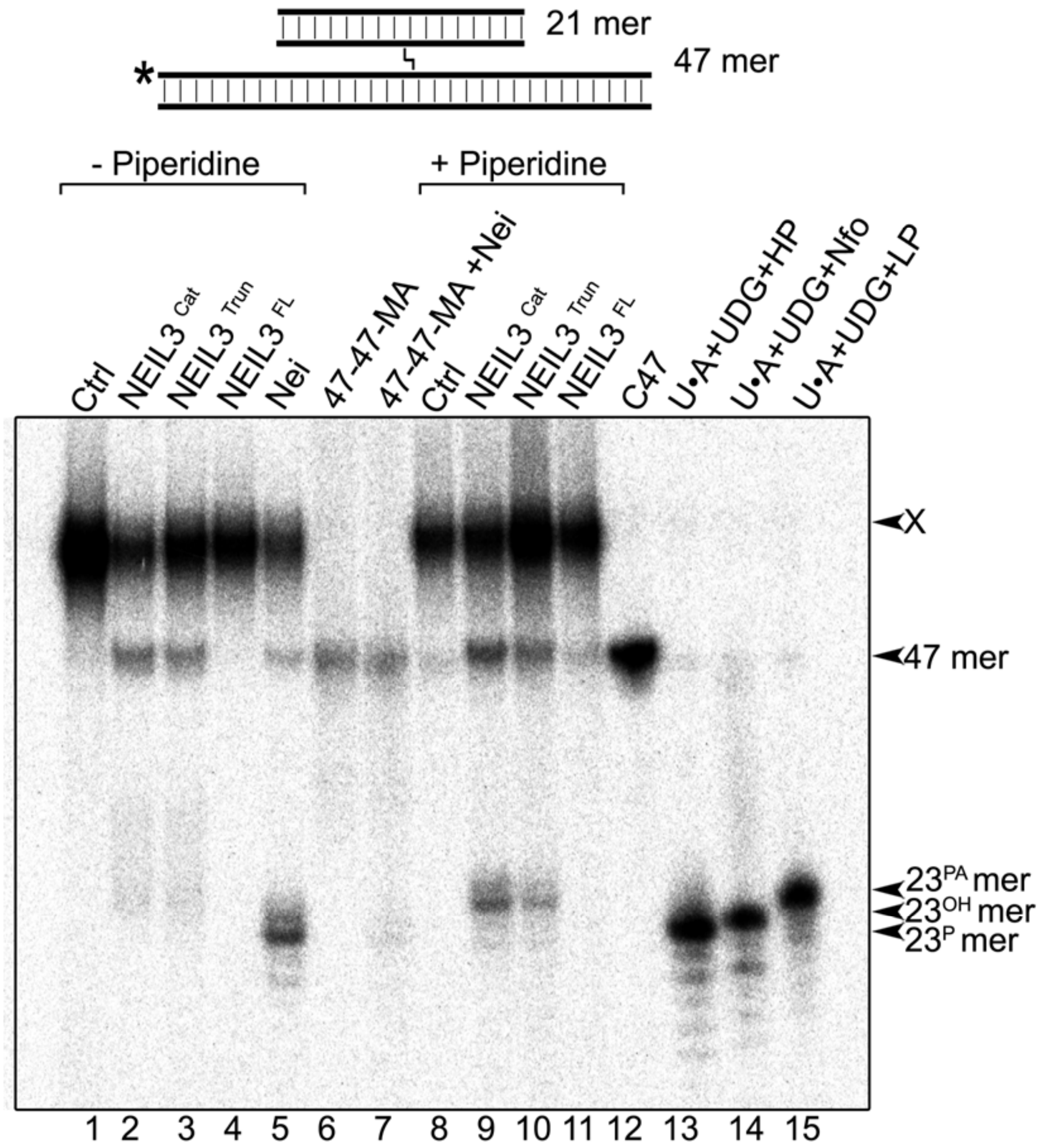

Supplementary Figure S8. **Action of Nei-like DNA glycosylases upon four-stranded DNA structure containing single HMT-derived ICL**. Denaturing PAGE analysis of the reaction products. 10 nM 5'-[^{32}P]-labelled XL47•47*-21•21 was incubated for 1 hr at 37°C either with the 500 nM NEIL3^{Cat}, NEIL3^{Trun}, NEIL3^{FL} or with 20 nM Nei proteins. Lanes 1-7, no piperidine treatment; lane 1, control non-treated XL47•47*-21•21; lanes 2-4, as 1 but with NEIL3s; lane 5, as 1 but Nei; lane 6, 47 mer duplex containing HMT-derived MA; lane 7, as 6 but with Nei; lanes 8-11, as 1-4 but treated with light piperidine, lane 12, 47 mer size marker, C47; lanes 13-15, 47 mer U•A duplex treated with UDG/hot piperidine, UDG/Nfo and UDG/light piperidine, respectively. Substrate and cleavage products sizes are indicated to the right of the gel. X" denotes ICL substrate, "47 mer" denotes 47 mer size marker, "23^{PA}", "23^{OH}" and "23^{P}" denote 23 mer fragments containing 3'-terminal PA, OH and P, respectively. For details see Materials and Methods.

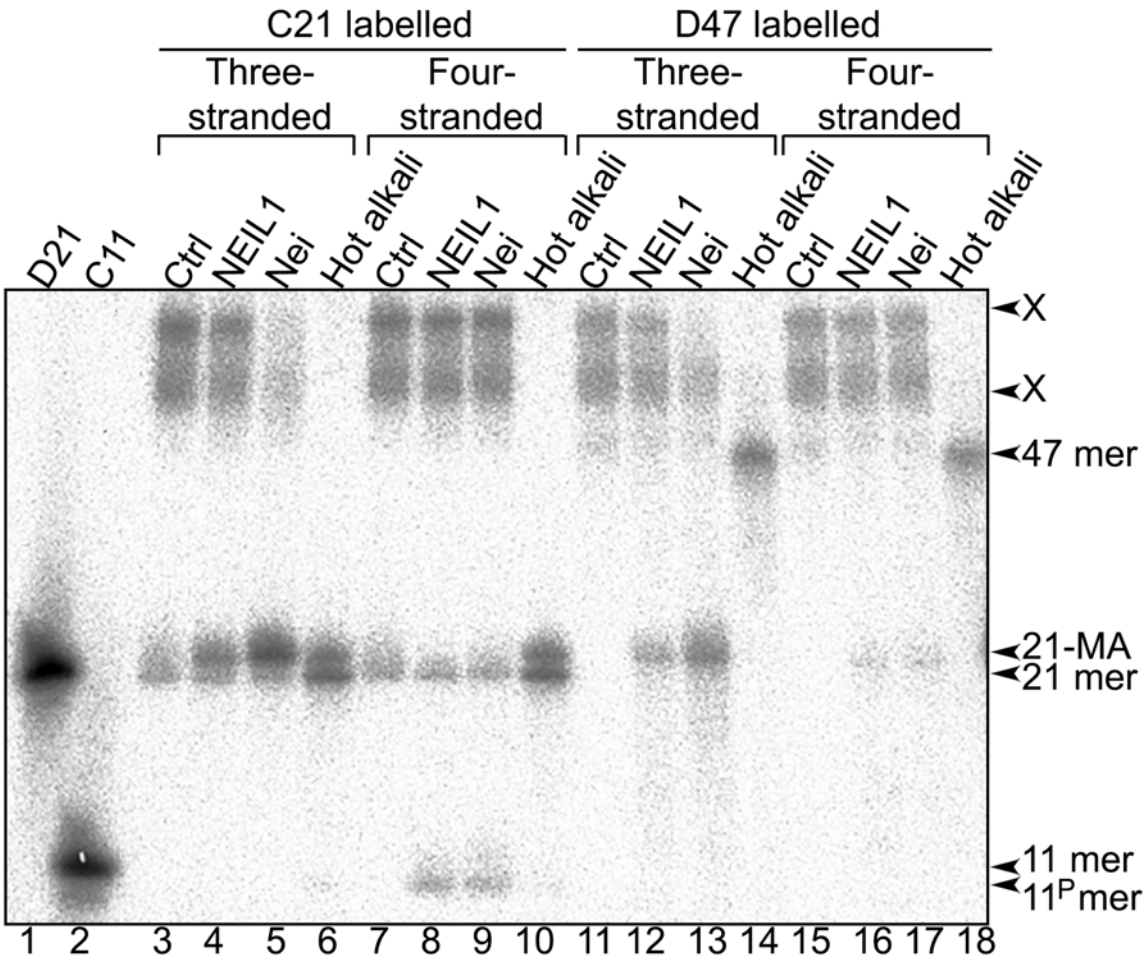

Supplementary Figure S9. Action of NEIL1 and Nei upon three- and four-stranded DNA structures containing single 8-MOP-derived ICL. 10 nM 5'-[^{32}P]-labelled XL47•47-21*, XL47•47*-21, XL47•47-21*•21 and XL47•47*-21•21 were incubated with 20 nM Nei or NEIL1 for 1 hr at 37°C. The products of reaction were analyzed on denaturing PAGE. Lane 1, 21 mer size marker; lane 2, 11 mer size marker; lane 3, control XL47•47-21*; lane 4, as 3 but NEIL1; lane 5, as 1 but Nei; lane 6, as 1 but hot hot alkali treatment; lanes 7-10, same as 3-6, but with XL47•47-21*•21; lanes 11-14, same as 3-6, but with XL47•47*-21; lanes 15-18, same as 3-6 but with XL47•47*-21•21. Substrate and cleavage products sizes are indicated to the right of the gel. "X" denotes substrate, "47 mer" denotes alkaline cleavage product containing either MA or regular thymine residue, "21-MA" denotes 21 mer fragment containing 8-MOP-derived MA. "21 mer" denotes 21 mer size marker, and "11 mer" denotes 11 mer size marker. For details see Materials and Methods.

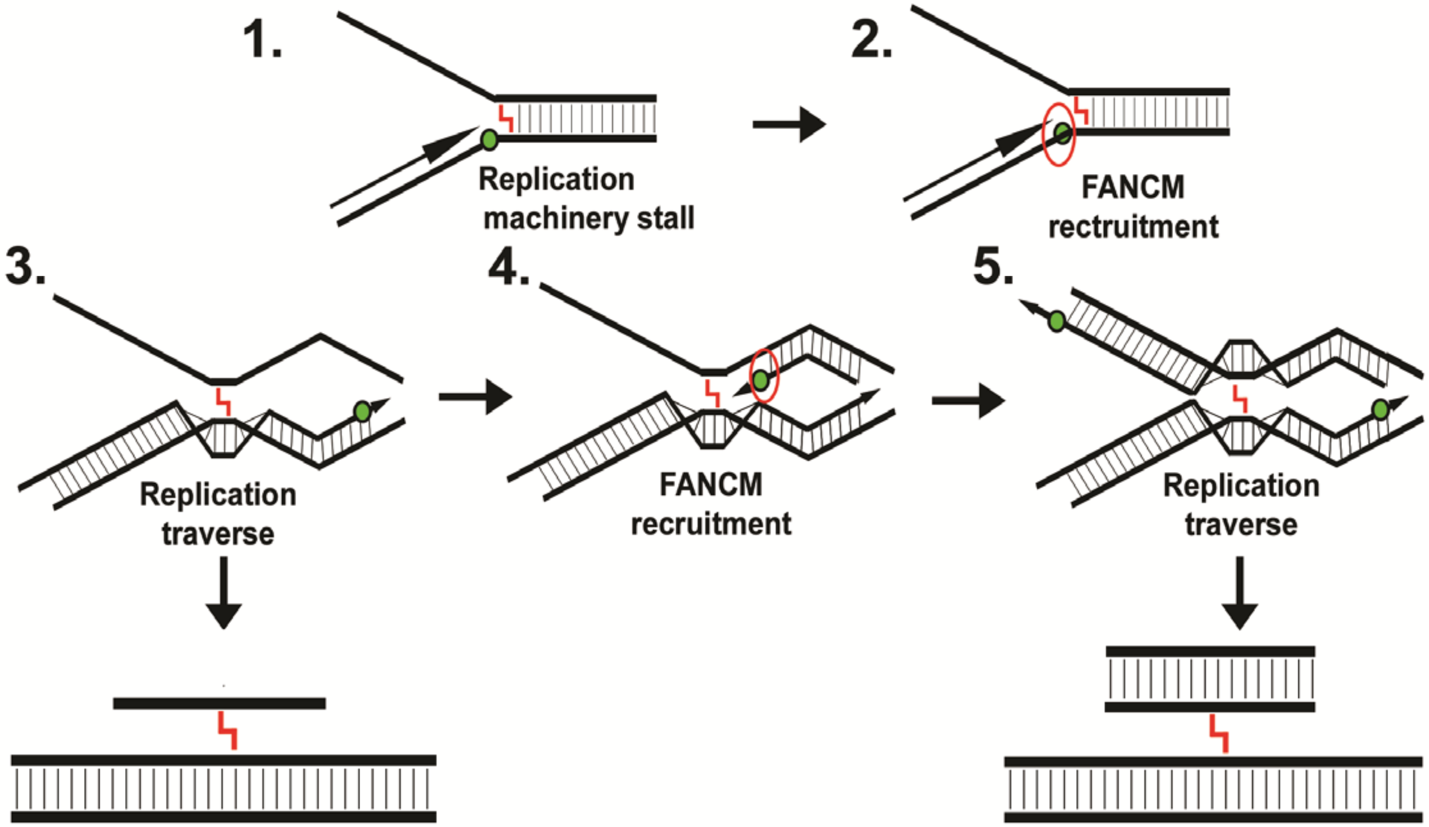

Supplementary Figure S10. **The putative mechanism of the FANCM-mediated DNA replication fork bypass of ICL and formation three- and four-stranded DNA structures with unrepaired crosslink**. (1) The leading strand synthesis reaches an ICL and causes replication fork stall; (2) The stalled replication fork recruits FANCM; (3) FANCM promotes leading strand synthesis bypass across the ICL generating a three-stranded DNA structure with an unrepaired ICL; (4) FANCM-promoted replication fork traverse/bypass of the ICL creates a new replication fork downstream of the lesion. This in turn initiates lagging strand synthesis which stalls at the ICL and recruits FANCM for a second time. (5) FANCM promotes lagging strand synthesis bypass across the ICL generating a four-stranded DNA structure with an unrepaired ICL.
